# Supplementary material for: Daytime napping, sleep duration and serum C reactive protein: a population-based cohort study
Source: BMJ Open. 2014 Nov 11;4(11):e006071. doi: 10.1136/bmjopen-2014-006071 (PMC4244397; doi:10.1136/bmjopen-2014-006071)
Supplement: Author's manuscript [file bmjopen-2014-006071.draft_revisions.pdf]

Daytime napping, sleep duration and serum C-reactive protein: a population-based cohort study

Journal:	*BMJ Open*
Manuscript ID:	bmjopen-2014-006071
Article Type:	Research
Date Submitted by the Author:	08-Jul-2014
Complete List of Authors:	Leng, Yue; University of Cambridge, Department of Public Health and Primary Care<br>Ahmadi-Abhari, Sara; University of Cambridge, Department of Public Health and Primary Care<br>Wainwright, Nicholas ; University of Cambridge, UK,<br>Cappuccio, Francesco; University of Warwick, Warwick Medical School, Clinical Sciences Research Institute<br>Surtees, Paul; University of Cambridge, Department of Public Health and Primary Care<br>Luben, Robert; University of Cambridge, Department of Public Health and Primary Care<br>Brayne, Carol; University of Cambridge, Institute of Public Health<br>Khaw, Kay-Tee; University of Cambridge, Department of Public Health and Primary Care
**Primary Subject Heading**:	Epidemiology
Secondary Subject Heading:	Public health
Keywords:	Sleep, Napping, C-reactive protein, Inflammation, Population

SCHOLARONE™ Manuscripts

Daytime napping, sleep duration and serum C-reactive protein: a population-based cohort study

Yue Leng, M,Phil[1*], Sara Ahmadi-Abhari, MD[1], Nick W.J. Wainwright, PhD[1], Francesco P. Cappuccio, FRCP, FAHA[2], Paul G. Surtees, PhD[1], Robert Luben, MSc[1], Carol Brayne, MD, FRCP [3], Kay-Tee Khaw, MD, FRCP[1]

Affiliations
[1] Department of Public Health and Primary Care, Strangeways Research Laboratory, University of Cambridge, Cambridge, UK
[2] Division of Mental Health & Wellbeing, Warwick Medical School, University of Warwick, Coventry, UK
[3] Department of Public Health and Primary Care, Institute of Public Health, University of Cambridge, Cambridge, UK

For peer review only

Word count: 2975 (Main text, excluding title page, abstract, references, figures and tables);

[*] Address for correspondence: Yue Leng, Department of Public Health and Primary Care, Strangeways Research Laboratory, University of Cambridge, Cambridge CB1 8RN, UK (Email: yl411@medschl.cam.ac.uk; Tel: +44(0)1223748686)

For peer review only - http://bmjopen.bmj.com/site/about/guidelines.xhtml

ABSTRACT

Objectives: To explore whether daytime napping and sleep duration are linked to serum C-reactive protein (CRP), a pro-inflammatory marker, in an older-aged British population.

Design: Population-based prospective cohort study

Setting: European Prospective Investigation into Cancer and Nutrition (EPIC)-Norfolk study.

Participants: A total of 5018 men and women aged 48-92 years reported their sleep habits and had serum CRP levels measured.

Outcome and measures: CRP was measured (mg/l) during 2006-2011 in fresh blood samples using high-sensitivity methods. Participants reported napping habits during 2002-2004, and reported sleep quantity during 2006-2007. Multivariable linear regression models were used to examine the association between napping and log-transformed CRP, and geometric mean CRP levels were calculated.

Results: After adjustment for age and sex, those who reported napping had 10% higher CRP levels compared to those not napping. The association was attenuated but remained borderline significant [β= 0.05 (95% CI: 0.00, 0.10)] after further adjustment for social class, education, marital status, body mass index, physical activity, smoking, alcohol intake, self-reported health, pre-existing diseases, systolic blood pressure, hypnotic drug use, depression and in women only hormone replacement therapy use. The geometric means (95%CI) of CRP levels were 2.38 (2.29-2.47) mg/l and 2.26 (2.21-2.32) mg/l for those who reported napping and no

For peer review only - http://bmjopen.bmj.com/site/about/guidelines.xhtml

For peer review only

napping, respectively. A U-shaped association was observed between time spent in bed at night and CRP levels, and night time sleep duration was not associated with serum CRP levels. The association between napping and CRP was stronger for older participants, and amongst extremes of time spent in bed at night.

Conclusions: Daytime napping was associated with increased CRP levels in an older-aged British population. Further studies are needed to determine whether daytime napping is a cause for systemic inflammation, or if it is a symptom or consequence of underlying health problems.

Keywords: *Sleep; Napping; C-reactive protein; Inflammation; Population*

For peer review only

For peer review only - http://bmjopen.bmj.com/site/about/guidelines.xhtml

Article summary

Article focus:

- Daytime napping has been associated with cardiovascular disease and mortality, but the biological implication of napping is unclear.
- To our knowledge, this is the first population-based study to examine the association between daytime napping and serum CRP levels.
- We also studied both time spent in bed at night and sleep duration in relation to CRP levels.

Key messages:

- Daytime napping was associated with 10% higher CRP levels, independent of age and sex.
- The association was more pronounced in women, older participants, and amongst extremes of time spent in bed at night.
- Sleep duration was not associated with CRP levels.

Strengths and limitations:

- Our study benefits from a large population-based sample, measures of both daytime and night time sleep, and a wide range of covariates available for adjustment.
- Sleep habits were self-reported
- The purpose and duration of napping was not reported, and sleep apnea was not measured.

For peer review only - http://bmjopen.bmj.com/site/about/guidelines.xhtml

Introduction

Recently there has been growing interest in studying the health implications of daytime napping. Although the benefits of napping on wakefulness performance have been widely documented, especially among shift workers, [1] a few studies also reported an increased health risk associated with daytime napping. Specifically, daytime napping has been associated with increased mortality, [2,3] cardiovascular, [4]and diabetes risk. [5] We have recently found a 32% increased mortality risk among those who napped for ≥1h/ day in a middle- to older- aged English population. [6] While it is not yet clear whether daytime napping is a cause or symptom of the increased health risk, examination of the physiological correlations of napping will help to gain understanding of the napping-health relationship.

One important proposed pathway for the association between sleep and health outcomes is through inflammation.[7] C-reative protein (CRP), a general marker for inflammation, was first suggested to increase after sleep depreviation in an experimental study.[8] Since then, there have been increasing numbers of observational studies which produced mixed findings on the association. Long sleep duration has been associated with increasing CRP among men,[9] women,[10] as well as in the sexes combined sample,[11,12] with long sleep being brought up as a potential marker of underlying inflammatory illness. [11] By contrast, Miller et al [13] found an association between short sleep duration and increased CRP only in women. Taheri et al suggested no significant association between CRP levels and sleep duration in the Wisconsin Sleep Cohort Study.[14]

For peer review only - http://bmjopen.bmj.com/site/about/guidelines.xhtml

Despite the growing interest into the inflammatory correlates of night time sleep, there has been limited evidence on the physiological effects of daytime napping. Experimental study has reported a beneficial effect of napping on immune cells following acute sleep restriction.[15] However, habitual daytime napping as a lifestyle might relate to different physiological effects compared to napping as a form of recovery sleep, and understanding the link between habitual napping and inflammation could help to clarify the association observed for napping and the increased mortality risk. Therefore we set out to examine the associations between daytime napping and serum CRP levels in the European Prospective Investigation into Cancer (EPIC)-Norfolk cohort study. In addition, we have previously reported the different implications between sleep duration and time spent in bed, [16] so confirmatory analysis was also conducted to test the associations separately for sleep duration and time spent in bed at night. Given the gender disparity suggested by some previous studies,[14, 18] we explored sex differences in the associations.

Methods

Participants

The design and study methods of EPIC-Norfolk have been described previously.[18]Briefly, 25,639 men and women were recruited into the EPIC-Norfolk study during 1993-1997 using general practice age-sex registers, and attended the baseline health check. These participants were then followed up for two further health examinations from 1996 to 2000 and from 2006 to 2011. In between these health examinations, participants were sent questionnaires for completion and returned by

For peer review only - http://bmjopen.bmj.com/site/about/guidelines.xhtml

post. The Norwich District Ethics Committee approved the study and all participants gave signed informed consent.

Sleep Measures

Habitual daytime napping was ascertained during 2002-2004, by asking participants the question "Do you normally take a nap during the day?".

"Night time sleep duration" and "Time spent in bed at night" were ascertained during 2006-2007, by asking participants "On average, about how many hours have you slept each night?"; "At what time do you normally get up?" and "At what time do you normally go to bed?". "Time spent in bed" was derived from the differences between rise time and bedtime, and as a weighted mean measure of weekday and weekend times [5/7*(time on a week- day)+ 2/7*(time on a weekend day)].

CRP

CRP was measured (mg/l) at the third health examination (2006-2011) in fresh blood samples using high-sensitivity methods using the Siemens Dimension clinical chemistry analyzer (Siemens Dimension clinical chemistry analyzer, Newark, Delaware, US).

Covariates

Covariates measured closer in time to each measure of sleep were chosen and included in the models for the corresponding sleep measures. The covariates were chosen as a priori based on their link with CRP and sleep.[16,19] Social class (professionals, managerial and technical occupations, skilled workers subdivided into non-manual and manual, partly skilled workers and unskilled manual workers) and

For peer review only - http://bmjopen.bmj.com/site/about/guidelines.xhtml

education (highest qualification attained: no qualifications, educated to age 16, educated to age 18, and educated to degree level) were assessed at the baseline questionnaire. Body Mass Index (BMI; weight in kilograms divided by height in meters squared) and Systolic Blood Pressure (SBP) (mmHg) were objectively assessed through the health examination. The other covariates reported by questionnaires included: marital status (single, married, widowed, separated and divorced), physical activity (inactive, moderately inactive, moderately active and active[20]), smoking status(current, former and non-smokers), alcohol intake (units of alcohol drunk per week), self-reported general health (excellent, good, moderate or poor), Major Depressive Disorder (MDD) (Yes/No), pre-existing diseases (including self-reported stroke, myocardial infarction, diabetes, cancer, asthma, bronchitis, and emphysema), use of hypnotic drugs (Yes/No), and Hormone Replacement Therapy (HRT) use.

Statistical analysis

Analysis was restricted to those participants with complete data on all covariates. A total of 5018 participants had complete data on CRP and at least one of the sleep measures and were included in the analysis.

The CRP values were natural-log transformed ($\log_e$ CRP) to approximate a normal distribution. Due to the U-shaped relationship reported by previous studies on sleep duration and health risk,[22,23] and in order to retain sufficient numbers, sleep duration was categorized into three categories (<6, 6-8, and >8 hours) and time spent in bed was categorized into four categories (<6, 6-8, 8-10, and >10 hours). Multivariable linear regression models using $\log_e$ CRP as the outcome were conducted to examine the associations between each sleep measure (daytime napping [N=4712], time spent

For peer review only - http://bmjopen.bmj.com/site/about/guidelines.xhtml

in bed [N=4476] and sleep duration [N=4795]) and CRP levels. For each sleep variable, results were presented: A. adjusted for age, sex; B. further adjusted for social class, education and marital status, BMI, physical activity, smoking and alcohol intake, depression, self-reported health, pre-existing diseases, SBP, hypnotic drug use and in women only postmenopausal HRT. The sleep measures were mutually adjusted in model B. Since the associations for sleep duration and time in bed might be non-linear (approximately U-shaped), the β coefficients (with 95% confidence intervals) were calculated with 6-8h being the reference groups for the examination of sleep quantitative measures. For analysis by napping habit, the no napping category was chosen as reference group. Results were presented for the whole sample and for men and women separately.

Geometric-mean (95% confidence interval, CI) serum CRP values (mg/l) were calculated by exponentiating crude and adjusted-least square mean (95% CI) $\log_e$ CRP values. Finally, subgroup analysis was conducted for the main sleep variable of interest, napping and time spent in bed at night, to explore the effects of potential effect modifiers (including age, sex, social class, smoking, BMI, physical activity and pre-existing diseases). These analyses were adjusted for age, sex, social class, education, marital status and BMI. The two by two interactions between napping habit, time in bed, and sleep duration were examined using the likelihood ratio test. Sensitivity analysis All statistical tests were two-sided, and a p value <0.05 was considered statistically significant. Analyses were implemented in STATA 12.0.

For peer review only - http://bmjopen.bmj.com/site/about/guidelines.xhtml

Results

Characteristics of the study sample (2267 men, 2751 women) are shown in table 1. The mean age of the participants was 69.5 (ranging from 48 to 92) years old. More than 60% of the sample came from non-manual social class, and 48.0% were former or current smokers. A total of 38.5% men and 23.1% women reported daytime napping. The average time people spent in bed every night was 8.6±0.8 h and the average sleep duration was 6.9±1.1 h. Serum CRP ranged from 0.1-116.6 mg/l, with a median of 2.0 mg/l (interquartile range 2.07 mg/l).

For peer review only

Table 1 Baseline characteristics of the EPIC-Norfolk participants

	Men	Women	Total
**Age** (years) ƚ	70.5 (8.0)	68.6 (7.9)	69.5 (8.0)
**Social class**			
Non-manual	1495 (66.5%)	1885 (69.3%)	3380 (68.0%)
Manual	753 (33.5%)	836 (30.7%)	1589 (32.0%)
**Education**			
Lower	686 (30.3%)	1148 (41.7%)	1834 (36.6%)
Higher	1580 (69.7%)	1602 (58.3%)	3182 (63.4%)
**Marital status**			
Single	60 (2.8%)	99 (3.8%)	159 (3.3%)
Married	1929 (90.4%)	2017 (77.6%)	3946 (83.4%)
Others	144 (6.8%)	484 (18.6%)	628 (13.3%)
**Smoking**			
Current smoker	147 (6.5%)	194 (7.1%)	341 (6.8%)
Former smoker	1178 (52.1%)	881 (32.1%)	2059 (41.2%)
Never smoked	936 (41.4%)	1666 (60.8%)	2602 (52.0%)
**Physical activity**			
Inactive	624 (29.0%)	677 (25.9%)	1301 (27.4%)
Moderately inactive	534 (24.9%)	877 (33.6%)	1411 (29.6%)
Moderately active	497 (23.1%)	613 (23.5%)	1110 (23.3%)
Active	493 (23.0%)	447 (17.1%)	940 (19.7%)
**BMI** (kg/m^2) ƚ	26.7 (3.1)	26.0 (4.0)	26.3 (3.6)
**SBP** (mmHg) ƚ	134.9 (16.9)	129.9 (17.3)	132.2 (17.3)
**Alcohol** (units)*	8.0 (3.0-15.0)	3.0 (1.5-8.0)	5.0 (2.0-11.0)
**Pre-existing diseases**			
No	2095 (97.5%)	2499 (98.9%)	4594 (98.2%)
Yes	53 (2.5%)	29 (1.1%)	82 (1.8%)
**Hypnotic use**			
No	2236 (98.6%)	2717 (98.8%)	4953 (98.7%)
Yes	31 (1.4%)	34 (1.2%)	65 (1.3%)
**MDD in the last year**			
No	1964 (97.1%)	2336 (94.3%)	4300 (95.6%)
Yes	58 (2.9%)	142 (5.7%)	200 (4.4%)
**Self-reported general health**			
Excellent	443 (19.7%)	564 (20.6%)	1007 (20.2%)
Good	1549 (68.8%)	1828 (66.8%)	3377 (67.7%)
Moderate	245 (10.9%)	332 (12.1%)	577 (11.6%)
Poor	15 (0.7%)	11 (0.4%)	26 (0.5%)
**Napping**			
No	1309 (61.5%)	1986 (76.9%)	3295 (69.9%)
Yes	819 (38.5%)	598 (23.1%)	1417 (30.1%)
**Time in bed** (h) ƚ	8.5 (0.9)	8.7 (0.8)	8.6 (0.8)
**Sleep duration** (h) ƚ	7.0 (1.1)	6.9 (1.1)	6.9 (1.1)
**CRP** (mg/l)*	1.9 (1.3-3.2)	2.0 (1.3-3.5)	2.0 (1.3-3.4)

Results were presented as ƚ mean (SD), * median (IQR) and the rest N(%). BMI: Body Mass Index; SBP: Systolic Blood Pressure; MDD: Major Depressive Disorder; CRP: C-reactive protein.

For peer review only

For peer review only - http://bmjopen.bmj.com/site/about/guidelines.xhtml

The unadjusted geometric means of CRP were 2.53 (2.44-2.63) mg/l and 2.20 (2.15-2.26) mg/l for those who reported napping and no napping, respectively. Table 2 shows differences in $\log_e$ CRP according to report of napping. After adjustment for age and sex, daytime napping was associated with 10% higher CRP levels [β= 0.10, 95%CI (0.05, 0.15), p<0.001]. With further adjustment for socio-economic status, health-related behaviors, physical health and night time sleep, the association was attenuated but remained borderline significant [β= 0.05, 95%CI (0.00, 0.10), p=0.048]. Table 3 shows the multivariable-adjusted geometric means of CRP. The geometric means of CRP levels were 2.38 (2.29-2.47) mg/l and 2.26 (2.21-2.32) mg/l for those who reported napping and no napping, respectively. When analyses were stratified by sex, the associations between the above napping measure and CRP levels were only significant among women.

The unadjusted geometric means of CRP for those who reported spending <6, 6-8, 8-10 and >10 hours in bed at night were 3.70 (2.48-5.53), 2.25 (2.15-2.35), 2.29 (2.23-2.35) and 2.84 (2.52-3.19) mg/l, respectively. After adjustment for age and sex, spending <6h and >10h in bed were associated with 51% and 17% increase in CRP levels, respectively (table 2). After full adjustments, long time spent in bed was associated with 12% increase in CRP levels while the association between short time in bed and $\log_e$ CRP was not significant. The geometric means of CRP levels among those who spent >10h and <6h in bed were 2.57 (2.30-2.87) and 3.00 (2.04-4.40) mg/l, respectively, higher than those who spent 6-8h in bed [2.28 (2.19-2.38) mg/l]. Night time sleep duration was not associated with $\log_e$ CRP. Repeating the analyses restricted to individuals with CRP levels below 10 mg/L did not change the results.

For peer review only - http://bmjopen.bmj.com/site/about/guidelines.xhtml

Table 2 Linear regression on sleep and $\log_e$ CRP in the EPIC-Norfolk cohort study

	All [β (95% CI)]		Men	[β (95% CI)]		Women	[β (95% CI)]	
	Model A	Model B		Model A	Model B		Model A	Model B
**Napping (4712)**			2128			2584		
No (n=3295)	Reference		1309			1986		
Yes (n=1417)	0.10***[0.05,0.15]	0.05*[0.00,0.10]	819	0.08*[0.01,0.15]	0.03[-0.03,0.10]	598	0.12***[0.06,0.19]	0.07*[0.00,0.13]
**Time in bed (4476)**			2036			2440		
<6h (n=13)	0.51*[0.11,0.91]	0.27[-0.11,0.66]	7	0.26[-0.30,0.82]	0.18[-0.37,0.73]	6	0.79**[0.22,1.37]	0.43[-0.12,0.97]
6-8h (n=1101)	Reference		620	Reference		481	Reference	
8-10h (n=3208)	-0.01[-0.06,0.04]	0[-0.05,0.05]	1342	-0.03[-0.10,0.04]	-0.01[-0.09,0.06]	1866	0[-0.07,0.07]	0.02[-0.05,0.09]
>10h (n=154)	0.17**[0.05,0.29]	0.12*[0.00,0.24]	67	0.18[-0.02,0.37]	0.14[-0.05,0.33]	87	0.17*[0.00,0.33]	0.11[-0.05,0.26]
P	0.001	0.09		0.11	0.35		0.01	0.23
**Sleep duration (4795)**			2173			2622		
<6h (n=1232)	0[-0.04,0.05]	-0.02[-0.07,0.02]	476	-0.01[-0.09,0.06]	-0.05[-0.13,0.02]	756	0.02[-0.04,0.08]	-0.01[-0.07,0.05]
6-8h (n=3154)	Reference		1483	Reference		1671	Reference	
>8h (n=409)	0.04[-0.03,0.12]	0[-0.07,0.08]	214	0.08[-0.03,0.19]	0.02[-0.08,0.13]	195	-0.01[-0.11,0.10]	-0.01[-0.11,0.09]
p	0.57	0.59		0.28	0.31		0.86	0.95

Model A: age, sex; Model B: age, sex, social class, education, marital status, BMI, physical activity, smoking, alcohol, self-reported health, pre-existing diseases, SBP, hypnotic use, major depressive disorder, and in women only HRT use. The sleep measures were mutually adjusted in model B. Results were presented as β coefficients (95%CI) of linear regression models using $\log_e$ CRP as the outcome.
*p<0.05 ** p<0.01 ***p<0.001

For peer review only

Table 3 Geometric means (95% CI) of CRP (mg/l) by sleep in the EPIC-Norfolk cohort study

	All		Men			Women		
	Model A	Model B		Model A	Model B		Model A	Model B
**Napping (4712)**			2128			2584		
No (n=3295)	2.23 [2.17, 2.28]***	2.26 [2.21, 2.32]*	1309	2.18 [2.09, 2.27]*	2.22 [2.13, 2.31]	1986	2.27 [2.20, 2.35]***	2.30 [2.23, 2.37]*
Yes (n=1417)	2.47 [2.37, 2.57]	2.38 [2.29, 2.47]	819	2.36 [2.24, 2.49]	2.29 [2.18, 2.41]	598	2.57 [2.43, 2.73]	2.45 [2.31,2.59]
**Time in bed (4476)**			2036			2440		
<6h (n=13)	3.82 [2.56, 5.69]*	3.00 [2.04, 4.40]	7	2.97 [1.70, 5.21]	2.73 [1.58, 4.72]	6	5.09 [2.87, 9.01]**	3.46 [2.01, 5.93]
6-8h (n=1101)	2.30 [2.20, 2.40]	2.28 [2.18, 2.38]	620	2.29 [2.16, 2.43]	2.27 [2.14, 2.41]	481	2.30 [2.16, 2.45]	2.28 [2.14, 2.42]
8-10h (n=3208)	2.27 [2.21, 2.33]	2.29 [2.23, 2.34]	1342	2.22 [2.14,2.32]	2.24 [2.15,2.33]	1866	2.31 [2.23, 2.38]	2.32 [2.25, 2.39]
>10h (n=154)	2.73 [2.43, 3.06]**	2.57 [2.30, 2.87]*	67	2.74 [2.28,3.28]	2.61[2.19,3.11]	87	2.72 [2.34, 3.17]*	2.50 [2.17, 2.89]
**Sleep duration (4795)**			2173			2622		
<6h (n=1232)	2.28 [2.18, 2.37]	2.24 [2.15, 2.33]	476	2.19 [2.05, 2.35]	2.14 [2.00, 2.28]	756	2.34 [2.23, 2.46]	2.30 [2.19, 2.41]
6-8h (n=3154)	2.27 [2.21, 2.33]	2.29 [2.24, 2.35]	1483	2.22 [2.14, 2.31]	2.26 [2.17, 2.34]	1671	2.30 [2.23, 2.38]	2.32 [2.25, 2.40]
>8h (n=409)	2.36 [2.20, 2.53]	2.30 [2.15, 2.46]	214	2.41 [2.18, 2.66]	2.30 [2.09, 2.54]	195	2.29 [2.07, 2.53]	2.29 [2.09, 2.52]

Model A: age, sex; Model B: age, sex, social class, education, marital status, BMI, physical activity, smoking, alcohol, self-reported health, pre-existing diseases, SBP, hypnotic use, major depressive disorder, and in women only HRT use. The sleep measures were mutually adjusted in model B. Results were presented as geometric means (95%CI) of CRP (mg/l).

For peer review only - http://bmjopen.bmj.com/site/about/guidelines.xhtml

Subgroup analysis investigated differences in these associations by age, sex, social class, smoking, BMI, physical activity and pre-existing diseases (Table 4). These data suggested that the association between napping and CRP levels was stronger for those who were older as compared to those who were younger (p for interaction = 0.007). In addition, the association was more pronounced for extremes of time spent in bed at night (p for interaction=0.01) (Figure 1).

Table 4 The association between log $_e$ CRP and napping or time spent in bed at night, by subgroups

	**Napping** (Reference: No)		**Time spent in bed at night** (reference: 6-8h)					
	Yes	[β (95% CI)]	0-6h	[β (95% CI)]	8-10h	[β (95% CI)]	>10h	[β (95% CI)]
**Age**	**p for interaction=0.007**		p for interaction=0.66					
<70	0	[-0.06,0.06]	0.14	[-0.25,0.52]	0	[-0.06,0.06]	0.16*	[0.01,0.32]
>70	0.09**	[0.02,0.15]	0.02	[-0.59,0.63]	0.03	[-0.05,0.11]	0.12	[-0.04,0.28]
**Sex**	p for interaction=0.87		p for interaction=0.82					
Men	0.04	[-0.02,0.11]	0.07	[-0.40,0.53]	-0	[-0.08,0.06]	0.16	[-0.03,0.35]
Women	0.05	[-0.01,0.11]	0.15	[-0.33,0.62]	0.02	[-0.04,0.08]	0.1	[-0.04,0.23]
**Social class**	p for interaction=0.38		p for interaction=0.31					
Higher	0.03	[-0.03,0.08]	0.15	[-0.27,0.57]	0.01	[-0.05,0.06]	0.19**	[0.05,0.33]
Lower	0.07	[-0.01,0.14]	0.08	[-0.45,0.61]	0	[-0.08,0.09]	0	[-0.18,0.19]
**Smoking**	p for interaction=0.5		p for interaction=0.55					
Current or former smokers	0.01	[-0.05,0.08]	0.12	[-0.33,0.57]	-0	[-0.10,0.05]	0.12	[-0.04,0.29]
Never smoked	0.06*	[0.01,0.12]	0.11	[-0.37,0.60]	0.03	[-0.03,0.09]	0.11	[-0.04,0.26]
**BMI**	p for interaction=0.73		p for interaction=0.30					
Lower	0.06	[-0.00,0.12]	-0.15	[-0.76,0.46]	0.04	[-0.03,0.10]	0.18*	[0.02,0.34]
Higher	0.07*	[0.01,0.14]	0.36	[-0.05,0.77]	-0	[-0.10,0.04]	0.09	[-0.07,0.25]
**Physical activity**	p for interaction=0.48		p for interaction=0.41					
Inactive	0.06*	[0.00,0.12]	0.28	[-0.14,0.71]	0.02	[-0.04,0.08]	0.13	[-0.00,0.26]
Active	0.02	[-0.05,0.09]	-0.13	[-0.65,0.38]	-0	[-0.10,0.05]	0.08	[-0.14,0.29]
**Pre-existing diseases**	p for interaction=0.05		p for interaction=0.08					
Yes	0.05	[-0.25,0.35]	N/A		0.19	[-0.19,0.56]	0.81**	[0.21,1.41]
No	0.05*	[0.01,0.10]	0.11	[-0.23,0.45]	0.02	[-0.03,0.07]	0.08	[-0.04,0.20]

The model adjusted for age, sex, social class, education, marital status and BMI.
Results were presented as β coefficients of linear regression models using logCRP as the outcome.
*p<0.05 ** p<0.01 ***p<0.001

For peer review only - http://bmjopen.bmj.com/site/about/guidelines.xhtml

For peer review only

Discussion

Our findings from more than 4000 older-aged English adults suggest that daytime napping was independently associated with higher CRP levels. Those who reported napping had 10% higher CRP levels after adjustment for age and sex. The association was attenuated but remained statistically significant after adjustment for all the covariates. A U-shaped association was observed between time spent in bed at night and CRP levels, though only the effect of long time spent in bed was statistically significant. The napping-CRP associations were more pronounced amongst extremes of time spent in bed at night. No association was found between sleep duration and CRP levels.

To our knowledge, this is the first study to report an association between habitual daytime napping and inflammation. This gives biological insights to the health impact of daytime napping proposed by previous studies.[4, 24] Our study benefits from a large population-based sample, and a wide range of covariates available for adjustment. Although only moderate effect size was detected, this preliminary finding at population level provides an interesting perspective and implies the need for further physiological studies. There are several limitations. Similar to most previous population studies,[24, 25] daytime napping was self-reported as yes or no. It was suggested that power naps (naps< 30min) can be largely beneficial, [2,26]while naps>30min can cause sleep inertia and are not recommended. [26] Without detailed report of napping duration, we were unable to differentiate the effects of power naps from excessive napping. Other sleep disorders (e.g. sleep apnea) that might have led to daytime sleepiness were not assessed, so the possibility of residual confounding by these sleep disorders can not be ruled out. Moreover, reported sleep may be a marker

For peer review only - http://bmjopen.bmj.com/site/about/guidelines.xhtml

of distress levels,[27] which has been linked with CRP levels.[28] However, evaluation of sleep in the primary care setting relies on self-reported data from patients, and also the association remained after adjustment for MDD. Another limitation of this study is that associations are based on a single measurement of CRP. However, the intra-individual variability of CRP measurements is similar to that of other physiologic measures such as blood pressure and cholesterol levels, and a single measurement of CRP has been shown to predict a range of health outcomes.[30,31] Besides, previous study has reported no diurnal variation of CRP concentrations.[31] Notably, while the assessment of napping preceded the CRP measures, baseline CRP level was not measured and there is no information on changes in CRP over time. We were therefore unable to distinguish between napping as a risk factor for, or as an early marker of, increased inflammatory levels. Finally, the current analysis was restricted to the 5018 participants with complete data on CRP and at least one of the sleep measures. Compared to the other participants from the baseline cohort (N=25,639), the present sample were 4 years younger, more likely to be of higher social class and higher education levels. Therefore, our findings have limited generalizability to populations with lower socioeconomic status.

The present study suggested that daytime napping and long time spent in bed at night were associated with higher CRP levels. The observed effect size was similar to previous report on sleep duration and CRP levels, [10] and the increase in CRP levels associated with napping was similar to that associated with 5-year increase in age. [19] While there are no other studies with which to directly compare our findings, the current study is in line with existing evidence on the increased cardiovascular, metabolic and mortality risks among those who take naps, found both by other studies and in our study population.[6,7,33,34] Meanwhile, a Greek cohort study suggested that

For peer review only - http://bmjopen.bmj.com/site/about/guidelines.xhtml

siesta might help to reduce coronary mortality through a stress-releasing mechanism in healthy working men. [34] We also found the association between daytime napping and increased CRP levels only significant among participants over 70 years old when analysis was stratified by age. Daytime napping might have different implications for health among different age groups, and this needs to be considered by future studies. Several studies suggested that the association between sleep and inflammation was only observed in women.[14,18] Consistent with these findings, the association between napping and increased CRP levels was only significant in women, although test for interaction was not statistically significant. The reason for this is unclear, and the gender disparities of sleep physiology need further exploration.

Time spent in bed was rarely studied in relation to CRP concentrations, but long sleep duration has been associated with increased CRP levels.[10,12] Since epidemiology studies have used mixed ways to define sleep duration, the associations between long sleep and inflammation found by some previous studies might actually reflect the effects of long time spent in bed, which was associated with poor general health. [16] We found no association between sleep duration and CRP levels. This is consistent with findings from the Wisconsin Sleep Cohort Study, which showed no association between CRP and sleep duration, measured both objectively and subjectively.[14] Meanwhile, previous studies have found both short and long sleep duration to be associated with increased CRP levels.[13, 14] Recently, a longitudinal study suggested that each hour per night decrease in sleep duration was associated with 8.1% higher average levels of CRP over a 5-year period. [35] The different correlations with serum CRP levels between the two sleep quantity measures need to be considered by future studies. Interestingly, the association between napping and CRP levels was more pronounced amongst extremes of time in bed. It is possible that extremes of time

For peer review only - http://bmjopen.bmj.com/site/about/guidelines.xhtml

spent in bed at night partly reflected disturbed night-time sleep, and thereby added to the association for daytime napping. While the complex inter-relationship between night time and daytime sleep is yet to be confirmed, the present study suggested a need for a thorough consideration of both night time and daytime sleep in future studies.

It has been suggested that sleep deprivation is related to the activation of the autonomic nervous system and increased catecholamines, and subsequently stimulate the release of inflammatory mediators. [36]It is unclear biologically why daytime napping was associated with increased levels of inflammatory markers. One possible explanation is through a rise in blood pressure or heart rate upon awakening from a nap, which might be followed by increased endothelial shear stress and the production of inflammatory mediators. [37]Alternatively, daytime napping could be the consequence of unnoticed night time sleep disturbance (e.g. sleep apnea) or sleep deprivation, which have been linked with systemic inflammation. [36,38,39] Sleep apnea was not measured in the current study, but the association between daytime napping and serum CRP levels remained after adjustment for BMI and SBP, two strong correlates of sleep apnea.

In the United Kingdom, daytime napping is not part of the cultural norm. It is plausible that those who reported daytime napping and long time spent in bed may represent a population with underlying ill health, and inflammation could be an early sign of these health problems. Although the association between daytime napping and CRP concentrations remained after adjustment for pre-existing diseases, we did find it more pronounced among the older participants, and there might be more existing health problems among this population. Further studies are needed to determine

For peer review only - http://bmjopen.bmj.com/site/about/guidelines.xhtml

whether daytime napping is a cause for systemic inflammation, or if it is a symptom or consequence of disturbed sleep at night or underlying health problems.

Conclusions

In summary, we found for the first time that daytime napping was associated with increased CRP levels in a population-based older aged English cohort. The association between daytime napping and CRP levels was more pronounced among older people, and among those who spent extremes of time in bed at night. While the present study provides new insights into the biological effects of daytime napping, causality can not be established from an observational study. Daytime napping might be useful as an independent indicator of people who are at underlying health risk or who suffer from disturbed sleep at night. Future physiological studies are needed to gain better understanding of the association.

For peer review only

For peer review only - http://bmjopen.bmj.com/site/about/guidelines.xhtml

Acknowledgement

We thank all study participants, general practitioners and the EPIC-Norfolk study team for their contribution.

Funding statement: The design and conduct of the EPIC-Norfolk study and collection and management of the data was supported by programme grants from the Medical Research Council UK (G9502233, G0300128) and Cancer Research UK (C865/A2883). YL is supported by the Cambridge Overseas Trust. FPC leads the Sleep Health & Society Programme at the University of Warwick supported, in part, by the University of Warwick RDF and IAS. It has received funding by the NHS National Workforce Projects and the Economic & Social Research Council (ES/K002910/1).

Authors' contributions

The work presented here was carried out in collaboration between all authors. YL and SA analyzed these data and wrote the manuscript with co-authors. YL, SA, NWJW, FPC, PGS, CB and KTK discussed the analysis, interpretation and presentation of these data. RL performed all data management and record linkage. RL and KTK are on the management team of EPIC-Norfolk population study and contributed substantially to acquisition of data. KTK is a principal investigator in the EPIC-Norfolk study. All authors provided detailed comments on the draft, and revised the manuscript critically. All authors read and approved the final manuscript.

Competing interests

None

For peer review only

For peer review only - http://bmjopen.bmj.com/site/about/guidelines.xhtml

Data Sharing Statement:

No additional data available

For peer review only

For peer review only - http://bmjopen.bmj.com/site/about/guidelines.xhtml

References

1 Milner C, Cote KA. Benefits of napping in healthy adults: impact of nap length, time of day, age, and experience with napping. *J Sleep Res* 2009;**18**:272–81.

2 Bursztyn M, Stessman J. The siesta and mortality: twelve years of prospective observations in 70-year-olds. *Sleep* 2005;**28**:345–7.

3 Jung K-I, Song C-H, Ancoli-Israel S, *et al.* Gender differences in nighttime sleep and daytime napping as predictors of mortality in older adults: the Rancho Bernardo study. *Sleep Med* 2013;**14**:12–9.

4 Tanabe N, Iso H, Seki N, *et al.* Daytime napping and mortality, with a special reference to cardiovascular disease: the JACC study. *Int J Epidemiol* 2010;**39**:233–43.

5 Xu Q, Song Y, Hollenbeck A, *et al.* Day napping and short night sleeping are associated with higher risk of diabetes in older adults. *Diabetes Care* 2010;**33**:78–83.

6 Leng Y, Wainwright NWJ, Cappuccio FP, *et al.* Daytime Napping and the Risk of All-Cause and Cause-Specific Mortality: A 13-Year Follow-up of a British Population. *Am J Epidemiol* 2014;**179**:1115–24.

7 Mullington JM, Simpson NS, Meier-Ewert HK, *et al.* Sleep loss and inflammation. *Best Pract Res Clin Endocrinol Metab* 2010;**24**:775–84.

8 Meier-Ewert HK, Ridker PM, Rifai N, *et al.* Effect of sleep loss on C-reactive protein, an inflammatory marker of cardiovascular risk. *J Am Coll Cardiol* 2004;**43**:678–83.

9 Jackowska M, Kumari M, Steptoe A. Sleep and biomarkers in the English Longitudinal Study of Ageing: associations with C-reactive protein, fibrinogen, dehydroepiandrosterone sulfate and hemoglobin. *Psychoneuroendocrinology* 2013;**38**:1484–93.

10 Patel SR, Zhu X, Storfer-Isser A, *et al.* Sleep duration and biomarkers of inflammation. *Sleep* 2009;**32**:200–4.

11 Dowd JB, Goldman N, Weinstein M. Sleep duration, sleep quality, and biomarkers of inflammation in a Taiwanese population. *Ann Epidemiol* 2011;**21**:799–806.

12 Grandner MA, Buxton OM, Jackson N, *et al.* Extreme sleep durations and increased C-reactive protein: effects of sex and ethnoracial group. *Sleep* 2013;**36**:769–779E.

For peer review only - http://bmjopen.bmj.com/site/about/guidelines.xhtml

13 Miller MA, Kandala N-B, Kivimaki M, *et al.* Gender differences in the cross-sectional relationships between sleep duration and markers of inflammation: Whitehall II study. *Sleep* 2009;**32**:857–64.

14 Taheri S, Austin D, Lin L, *et al.* Correlates of serum C-reactive protein (CRP)--no association with sleep duration or sleep disordered breathing. *Sleep* 2007;**30**:991–6.

15 Faraut B, Boudjeltia KZ, Dyzma M, *et al.* Benefits of napping and an extended duration of recovery sleep on alertness and immune cells after acute sleep restriction. *Brain Behav Immun* 2011;**25**:16–24.

16 Leng Y, Wainwright NWJ, Cappuccio FP, *et al.* Self-reported sleep patterns in a British population cohort. *Sleep Med* 2014;**15**:295–302.

17 Suarez EC. Self-reported symptoms of sleep disturbance and inflammation, coagulation, insulin resistance and psychosocial distress: evidence for gender disparity. *Brain Behav Immun* 2008;**22**:960–8.

18 Day N, Oakes SA, Luben R, *et al.* EPIC-Norfolk: study design and characteristics of the cohort. *Br J Cancer* 1999;**80**:95–103.

19 Ahmadi-Abhari S, Luben RN, Wareham NJ, *et al.* Distribution and determinants of C-reactive protein in the older adult population: European Prospective Investigation into Cancer-Norfolk study. *Eur J Clin Invest* 2013;**43**:899–911.

20 Wareham NJ, Jakes RW, Rennie KL, *et al.* Validity and repeatability of a simple index derived from the short physical activity questionnaire used in the European Prospective Investigation into Cancer and Nutrition (EPIC) study. *Public Health Nutr* 2003;**6**:407–13.

21 Heslop P, Smith GD, Metcalfe C, *et al.* Sleep duration and mortality: The effect of short or long sleep duration on cardiovascular and all-cause mortality in working men and women. *Sleep Med* 2002;**3**:305–14.

22 Hublin C, Partinen M, Koskenvuo M, *et al.* Sleep and mortality: a population-based 22-year follow-up study. *Sleep* 2007;**30**:1245–53.

23 Bursztyn M. The siesta in the elderly: risk factor for mortality? *Arch Intern Med* 1999;**159**:1582–6.

24 Cohen-Mansfield J, Perach R. Sleep duration, nap habits, and mortality in older persons. *Sleep* 2012;**35**:1003–9.

25 Ruggiero JS, Redeker NS. Effects of Napping on Sleepiness and Sleep-Related Performance Deficits in Night-Shift Workers: A Systematic Review. *Biol Res Nurs* 2014;**16**:134–42.

26 Tassi P, Muzet A. Sleep inertia. *Sleep Med Rev* 2000;**4**:341–53.

For peer review only

27 Lavie P. Self-reported sleep duration--what does it mean? *J Sleep Res* 2009;**18**:385–6.

28 Wium-Andersen MK, Ørsted DD, Nielsen SF, *et al.* Elevated C-reactive protein levels, psychological distress, and depression in 73, 131 individuals. *JAMA Psychiatry* 2013;**70**:176–84.

29 Allin KH, Bojesen SE, Nordestgaard BG. Baseline C-reactive protein is associated with incident cancer and survival in patients with cancer. *J Clin Oncol* 2009;**27**:2217–24.

30 Kaptoge S, Di Angelantonio E, Lowe G, *et al.* C-reactive protein concentration and risk of coronary heart disease, stroke, and mortality: an individual participant meta-analysis. *Lancet* 2010;**375**:132–40.

31 Meier-Ewert HK, Ridker PM, Rifai N, *et al.* Absence of diurnal variation of C-reactive protein concentrations in healthy human subjects. *Clin Chem* 2001;**47**:426–30.

32 Campos H, Siles X. Siesta and the risk of coronary heart disease: results from a population-based, case-control study in Costa Rica. *Int J Epidemiol* 2000;**29**:429–37.

33 Lam K-BH, Jiang CQ, Thomas GN, *et al.* Napping is associated with increased risk of type 2 diabetes: the Guangzhou Biobank Cohort Study. *Sleep* 2010;**33**:402–7.

34 Naska A, Oikonomou E, Trichopoulou A, *et al.* Siesta in healthy adults and coronary mortality in the general population. *Arch Intern Med* 2007;**167**:296–301.

35 Ferrie JE, Kivimäki M, Akbaraly TN, *et al.* Associations Between Change in Sleep Duration and Inflammation: Findings on C-reactive Protein and Interleukin 6 in the Whitehall II Study. *Am J Epidemiol* 2013;**178**:956–61.

36 Mullington JM, Haack M, Toth M, *et al.* Cardiovascular, inflammatory, and metabolic consequences of sleep deprivation. *Prog Cardiovasc Dis* 2009;**51**:294–302.

37 Chiu J-J, Usami S, Chien S. Vascular endothelial responses to altered shear stress: pathologic implications for atherosclerosis. *Ann Med* 2009;**41**:19–28.

38 Liukkonen T, Räsänen P, Ruokonen A, *et al.* C-reactive protein levels and sleep disturbances: observations based on the Northern Finland 1966 Birth Cohort study. *Psychosom Med* 2007;**69**:756–61.

39 Svensson M, Venge P, Janson C, *et al.* Relationship between sleep-disordered breathing and markers of systemic inflammation in women from the general population. *J Sleep Res* 2012;**21**:147–54.

For peer review only

For peer review only - http://bmjopen.bmj.com/site/about/guidelines.xhtml

Figure 1: Serum levels of C-reactive protein by napping and time in bed *

* Values are geometric mean CRP (mg/l) across categories adjusted for age, body mass index, physical activity, smoking, alcohol intake, social class, education, marital status, major depressive disorder, self-reported health, pre-existing diseases, systolic blood pressure, hypnotic drug use, and in women only, postmenopausal hormone replacement therapy. The sleep measures were mutually adjusted. Vertical bars represent 95% confidence intervals.

For peer review only

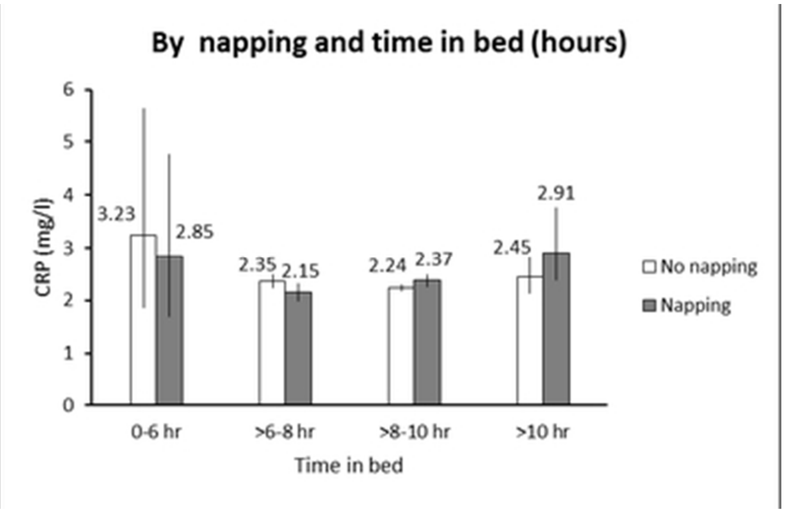

33x21mm (300 x 300 DPI)

STROBE 2007 (v4) checklist of items to be included in reports of observational studies in epidemiology*

Checklist for cohort, case-control, and cross-sectional studies (combined)

Section/Topic	Item #	Recommendation	Reported on page #
**Title and abstract**	1	(*a*) Indicate the study's design with a commonly used term in the title or the abstract	1
		(*b*) Provide in the abstract an informative and balanced summary of what was done and what was found	2
**Introduction**			
Background/rationale	2	Explain the scientific background and rationale for the investigation being reported	5
Objectives	3	State specific objectives, including any pre-specified hypotheses	6
**Methods**			
Study design	4	Present key elements of study design early in the paper	6-7
Setting	5	Describe the setting, locations, and relevant dates, including periods of recruitment, exposure, follow-up, and data collection	6-7
Participants	6	(*a*) *Cohort study*—Give the eligibility criteria, and the sources and methods of selection of participants. Describe methods of follow-up<br>*Case-control study*—Give the eligibility criteria, and the sources and methods of case ascertainment and control selection. Give the rationale for the choice of cases and controls<br>*Cross-sectional study*—Give the eligibility criteria, and the sources and methods of selection of participants	6-7
		(*b*) *Cohort study*—For matched studies, give matching criteria and number of exposed and unexposed<br>*Case-control study*—For matched studies, give matching criteria and the number of controls per case	
Variables	7	Clearly define all outcomes, exposures, predictors, potential confounders, and effect modifiers. Give diagnostic criteria, if applicable	7-8
Data sources/ measurement	8*	For each variable of interest, give sources of data and details of methods of assessment (measurement). Describe comparability of assessment methods if there is more than one group	7
Bias	9	Describe any efforts to address potential sources of bias	8-9
Study size	10	Explain how the study size was arrived at	8
Quantitative variables	11	Explain how quantitative variables were handled in the analyses. If applicable, describe which groupings were chosen and why	8-9
Statistical methods	12	(*a*) Describe all statistical methods, including those used to control for confounding	9
		(*b*) Describe any methods used to examine subgroups and interactions	9
		(*c*) Explain how missing data were addressed	8
		(*d*) *Cohort study*—If applicable, explain how loss to follow-up was addressed<br>*Case-control study*—If applicable, explain how matching of cases and controls was addressed	8

For peer review only

		Cross-sectional study—If applicable, describe analytical methods taking account of sampling strategy	
		(*e*) Describe any sensitivity analyses	9
**Results**			
Participants	13*	(a) Report numbers of individuals at each stage of study—eg numbers potentially eligible, examined for eligibility, confirmed eligible, included in the study, completing follow-up, and analysed	10
		(b) Give reasons for non-participation at each stage	
		(c) Consider use of a flow diagram	
Descriptive data	14*	(a) Give characteristics of study participants (eg demographic, clinical, social) and information on exposures and potential confounders	10
		(b) Indicate number of participants with missing data for each variable of interest	13
		(c) *Cohort study*—Summarise follow-up time (eg, average and total amount)	
Outcome data	15*	*Cohort study*—Report numbers of outcome events or summary measures over time	
		Case-control study—Report numbers in each exposure category, or summary measures of exposure	
		Cross-sectional study—Report numbers of outcome events or summary measures	8
Main results	16	(*a*) Give unadjusted estimates and, if applicable, confounder-adjusted estimates and their precision (eg, 95% confidence interval). Make clear which confounders were adjusted for and why they were included	12
		(*b*) Report category boundaries when continuous variables were categorized	
		(*c*) If relevant, consider translating estimates of relative risk into absolute risk for a meaningful time period	
Other analyses	17	Report other analyses done—eg analyses of subgroups and interactions, and sensitivity analyses	15
**Discussion**			
Key results	18	Summarise key results with reference to study objectives	16
Limitations	19	Discuss limitations of the study, taking into account sources of potential bias or imprecision. Discuss both direction and magnitude of any potential bias	16-17
Interpretation	20	Give a cautious overall interpretation of results considering objectives, limitations, multiplicity of analyses, results from similar studies, and other relevant evidence	17-19
Generalisability	21	Discuss the generalisability (external validity) of the study results	17
**Other information**			
Funding	22	Give the source of funding and the role of the funders for the present study and, if applicable, for the original study on which the present article is based	21

*Give information separately for cases and controls in case-control studies and, if applicable, for exposed and unexposed groups in cohort and cross-sectional studies.

Note: An Explanation and Elaboration article discusses each checklist item and gives methodological background and published examples of transparent reporting. The STROBE checklist is best used in conjunction with this article (freely available on the Web sites of PLoS Medicine at http://www.plosmedicine.org/, Annals of Internal Medicine at http://www.annals.org/, and Epidemiology at http://www.epidem.com/). Information on the STROBE Initiative is available at www.strobe-statement.org.

For peer review only

BMJ Open

Daytime napping, sleep duration and serum C-reactive protein: a population-based cohort study

Journal:	*BMJ Open*
Manuscript ID:	bmjopen-2014-006071.R1
Article Type:	Research
Date Submitted by the Author:	04-Sep-2014
Complete List of Authors:	Leng, Yue; University of Cambridge, Department of Public Health and Primary Care<br>Ahmadi-Abhari, Sara; University of Cambridge, Department of Public Health and Primary Care<br>Wainwright, Nicholas; University of Cambridge, UK, Department of Public Health and Primary Care, Strangeways Research Laboratory,<br>Cappuccio, Francesco; University of Warwick, Warwick Medical School, Clinical Sciences Research Institute<br>Surtees, Paul; University of Cambridge, Department of Public Health and Primary Care<br>Luben, Robert; University of Cambridge, Department of Public Health and Primary Care<br>Brayne, Carol; University of Cambridge, Institute of Public Health<br>Khaw, Kay-Tee; University of Cambridge, Department of Public Health and Primary Care
**Primary Subject Heading**:	Epidemiology
Secondary Subject Heading:	Public health
Keywords:	Sleep, Napping, C-reactive protein, Inflammation, Population

SCHOLARONE™
Manuscripts

Daytime napping, sleep duration and serum C-reactive protein: a population-based cohort study

Yue Leng, M,Phil[1*], Sara Ahmadi-Abhari, MD[1], Nick W.J. Wainwright, PhD[1], Francesco P. Cappuccio, FRCP, FAHA[2], Paul G. Surtees, PhD[1], Robert Luben, MSc[1], Carol Brayne, MD, FRCP [3], Kay-Tee Khaw, MD, FRCP[1]

Affiliations
[1] Department of Public Health and Primary Care, Strangeways Research Laboratory, University of Cambridge, Cambridge, UK
[2] Division of Mental Health & Wellbeing, Warwick Medical School, University of Warwick, Coventry, UK
[3] Department of Public Health and Primary Care, Institute of Public Health, University of Cambridge, Cambridge, UK

For peer review only

Word count: 3195 (Main text, excluding title page, abstract, references, figures and tables);

[*] Address for correspondence: Yue Leng, Department of Public Health and Primary Care, Strangeways Research Laboratory, University of Cambridge, Cambridge CB1 8RN, UK (Email: yl411@medschl.cam.ac.uk; Tel: +44(0)1223748686)

For peer review only - http://bmjopen.bmj.com/site/about/guidelines.xhtml

ABSTRACT

Objectives: To explore whether daytime napping and sleep duration are linked to serum C-reactive protein (CRP), a pro-inflammatory marker, in an older-aged British population.

Design: Cross-sectional study

Setting: European Prospective Investigation into Cancer and Nutrition (EPIC)-Norfolk study.

Participants: A total of 5018 men and women aged 48-92 years reported their sleep habits and had serum CRP levels measured.

Outcome and measures: CRP was measured (mg/l) during 2006-2011 in fresh blood samples using high-sensitivity methods. Participants reported napping habits during 2002-2004, and reported sleep quantity during 2006-2007. Multivariable linear regression models were used to examine the association between napping and log-transformed CRP, and geometric mean CRP levels were calculated.

Results: After adjustment for age and sex, those who reported napping had 10% higher CRP levels compared to those not napping. The association was attenuated but remained borderline significant [β= 0.05 (95% CI: 0.00, 0.10)] after further adjustment for social class, education, marital status, body mass index, physical activity, smoking, alcohol intake, self-reported health, pre-existing diseases, systolic blood pressure, hypnotic drug use, depression and in women only hormone replacement therapy use. The geometric means (95%CI) of CRP levels were 2.38 (2.29-2.47) mg/l and 2.26 (2.21-2.32) mg/l for those who reported napping and no

For peer review only

napping, respectively. A U-shaped association was observed between time spent in bed at night and CRP levels, and night time sleep duration was not associated with serum CRP levels. The association between napping and CRP was stronger for older participants, and amongst extremes of time spent in bed at night.

Conclusions: Daytime napping was associated with increased CRP levels in an older-aged British population. Further studies are needed to determine whether daytime napping is a cause for systemic inflammation, or if it is a symptom or consequence of underlying health problems.

Keywords: *Sleep; Napping; C-reactive protein; Inflammation; Population*

For peer review only

For peer review only - http://bmjopen.bmj.com/site/about/guidelines.xhtml

Article summary

Article focus:

- Daytime napping has been associated with cardiovascular disease and mortality, but the biological implication of napping is unclear.
- To our knowledge, this is the first population-based study to examine the association between daytime napping and serum CRP levels.
- We also studied both time spent in bed at night and sleep duration in relation to CRP levels.

Key messages:

- Daytime napping was associated with 10% higher CRP levels, independent of age and sex.
- The association was more pronounced in women, older participants, and amongst extremes of time spent in bed at night.
- Sleep duration was not associated with CRP levels.

Strengths and limitations:

- Our study benefits from a large population-based sample, measures of both daytime and night time sleep, and a wide range of covariates available for adjustment.
- Sleep habits were self-reported
- The purpose and duration of napping was not reported, and sleep apnea was not measured.

For peer review only - http://bmjopen.bmj.com/site/about/guidelines.xhtml

Introduction

Recently there has been growing interest in studying the health implications of daytime napping. Although the benefits of napping on wakefulness performance have been widely documented, especially among shift workers, [1] a few studies also reported an increased health risk associated with daytime napping. Specifically, daytime napping has been associated with increased mortality, [2,3] cardiovascular, [4]and diabetes risk. [5] We have recently found a 32% increased mortality risk among those who napped for ≥1h/ day in a middle- to older- aged English population. [6] While it is not yet clear whether daytime napping is a cause or symptom of the increased health risk, examination of the physiological correlations of napping will help to gain understanding of the napping-health relationship.

One important proposed pathway for the association between sleep and health outcomes is through inflammation.[7] C-reative protein (CRP), a general marker for inflammation, was first suggested to increase after sleep depreviation in an experimental study.[8] Since then, there have been increasing numbers of observational studies which produced mixed findings on the association. Long sleep duration has been associated with increasing CRP among men,[9] women,[10] as well as in the sexes combined sample,[11,12] with long sleep being brought up as a potential marker of underlying inflammatory illness. [11] By contrast, Miller et al [13] found an association between short sleep duration and increased CRP only in women. Taheri et al suggested no significant association between CRP levels and sleep duration in the Wisconsin Sleep Cohort Study.[14]

Despite the growing interest into the inflammatory correlates of night time sleep, there has been limited evidence on the physiological effects of daytime napping.

For peer review only - http://bmjopen.bmj.com/site/about/guidelines.xhtml

Experimental study has reported a beneficial effect of napping on immune cells following acute sleep restriction.[15] However, habitual daytime napping as a lifestyle might relate to different physiological effects compared to napping as a form of recovery sleep, and understanding the link between habitual napping and inflammation could help to clarify the association observed for napping and the increased mortality risk. Moreover, a number of changes in sleep from middle to older adulthood have been reported, including an advanced circadian pacemaker, decreased sleep efficiency and increased daytime sleepiness. [16,17,18] Therefore we set out to examine the associations between daytime napping and serum CRP levels in the European Prospective Investigation into Cancer (EPIC)-Norfolk cohort, a middle- to older- aged British population . In addition, we have previously reported the different implications between sleep duration and time spent in bed, [19] so confirmatory analysis was also conducted to test the associations separately for sleep duration and time spent in bed at night. Given the gender disparity suggested by some previous studies,[14, 18] we explored sex differences in the associations.

Methods

Participants

The design and study methods of EPIC-Norfolk have been described previously.[21]Briefly, 25,639 men and women were recruited into the EPIC-Norfolk study during 1993-1997 using general practice age-sex registers, and attended the baseline health check. These participants were then followed up for two further health examinations from 1996 to 2000 and from 2006 to 2011. In between these health examinations, participants were sent questionnaires for completion and returned by

For peer review only - http://bmjopen.bmj.com/site/about/guidelines.xhtml

post. The Norwich District Ethics Committee approved the study and all participants gave signed informed consent.

Sleep Measures

Habitual daytime napping was ascertained during 2002-2004, by asking participants the question “Do you normally take a nap during the day?”.

“Night time sleep duration” and “Time spent in bed at night” were ascertained during 2006-2007, by asking participants “On average, about how many hours have you slept each night?”; “At what time do you normally get up?” and “At what time do you normally go to bed?”. “Time spent in bed” was derived from the differences between rise time and bedtime, and as a weighted mean measure of weekday and weekend times [5/7*(time on a week- day)+ 2/7*(time on a weekend day)].

CRP

CRP was measured (mg/l) at the third health examination (2006-2011) in fresh blood samples using high-sensitivity methods using the Siemens Dimension clinical chemistry analyzer (Siemens Dimension clinical chemistry analyzer, Newark, Delaware, US), with between batch CV values of 3.1% at 7.86 mg/l, 3.7% at 88.75 mg/l.

Covariates

Covariates measured closer in time to each measure of sleep were chosen and included in the models for the corresponding sleep measures. The covariates were chosen as a priori based on their link with CRP and sleep.[19,22] Social class (professionals, managerial and technical occupations, skilled workers subdivided into

For peer review only - http://bmjopen.bmj.com/site/about/guidelines.xhtml

non-manual and manual, partly skilled workers and unskilled manual workers) and education (highest qualification attained: no qualifications, educated to age 16, educated to age 18, and educated to degree level) were assessed at the baseline questionnaire. Body Mass Index (BMI; weight in kilograms divided by height in meters squared) and Systolic Blood Pressure (SBP) (mmHg) were objectively assessed through the health examination. The other covariates reported by questionnaires included: marital status (single, married, widowed, separated and divorced), physical activity (inactive, moderately inactive, moderately active and active[23]), smoking status(current, former and non-smokers), alcohol intake (units of alcohol drunk per week), self-reported general health (excellent, good, moderate or poor), Major Depressive Disorder (MDD) (Yes/No), pre-existing diseases (including self-reported stroke, myocardial infarction, diabetes, cancer, asthma, bronchitis, and emphysema), use of hypnotic drugs (Yes/No), and Hormone Replacement Therapy (HRT) use.

Statistical analysis

Analysis was restricted to those participants with complete data on all covariates. A total of 5018 participants had complete data on CRP and at least one of the sleep measures and were included in the analysis.

The CRP values were natural-log transformed ($\log_e$ CRP) to approximate a normal distribution. Due to the U-shaped relationship reported by previous studies on sleep duration and health risk,[22,23] and in order to retain sufficient numbers, sleep duration was categorized into three categories (<6, 6-8, and >8 hours) and time spent in bed was categorized into four categories (<6, 6-8, 8-10, and >10 hours). Multivariable linear regression models using $\log_e$ CRP as the outcome were conducted to examine

For peer review only - http://bmjopen.bmj.com/site/about/guidelines.xhtml

the associations between each sleep measure (daytime napping [N=4712], time spent in bed [N=4476] and sleep duration [N=4795]) and CRP levels. For each sleep variable, results were presented: A. adjusted for age, sex; B. further adjusted for social class, education and marital status, BMI, physical activity, smoking and alcohol intake, depression, self-reported health, pre-existing diseases, SBP, hypnotic drug use and in women only postmenopausal HRT. The sleep measures were mutually adjusted in model B. Since the associations for sleep duration and time in bed might be non-linear (approximately U-shaped), the β coefficients (with 95% confidence intervals) were calculated with 6-8h being the reference groups for the examination of sleep quantitative measures. For analysis by napping habit, the no napping category was chosen as reference group. Results were presented for the whole sample and for men and women separately.

Geometric-mean (95% confidence interval, CI) serum CRP values (mg/l) were calculated by exponentiating crude and adjusted-least square mean (95% CI) $\log_e$ CRP values. Finally, subgroup analysis was conducted for the main sleep variable of interest, napping and time spent in bed at night, to explore the effects of potential effect modifiers (including age, sex, social class, smoking, BMI, physical activity and pre-existing diseases). These analyses were adjusted for age, sex, social class, education, marital status and BMI. The two by two interactions between napping habit, time in bed, and sleep duration were examined using the likelihood ratio test. Sensitivity analysis All statistical tests were two-sided, and a p value <0.05 was considered statistically significant. Analyses were implemented in STATA 12.0.

For peer review only - http://bmjopen.bmj.com/site/about/guidelines.xhtml

Results

Characteristics of the study sample (2267 men, 2751 women) are shown in table 1. The mean age of the participants was 69.5 (ranging from 48 to 92) years old. More than 60% of the sample came from non-manual social class, and 48.0% were former or current smokers. A total of 38.5% men and 23.1% women reported daytime napping. The average time people spent in bed every night was 8.6±0.8 h and the average sleep duration was 6.9±1.1 h. Serum CRP ranged from 0.1-116.6 mg/l, with a median of 2.0 mg/l (interquartile range 2.07 mg/l).

For peer review only

For peer review only - http://bmjopen.bmj.com/site/about/guidelines.xhtml

Table 1 Baseline characteristics of the EPIC-Norfolk participants

	Men	Women	Total
**Age** (years) ƚ	70.5 (8.0)	68.6 (7.9)	69.5 (8.0)
**Social class**			
Non-manual	1495 (66.5%)	1885 (69.3%)	3380 (68.0%)
Manual	753 (33.5%)	836 (30.7%)	1589 (32.0%)
**Education**			
Lower	686 (30.3%)	1148 (41.7%)	1834 (36.6%)
Higher	1580 (69.7%)	1602 (58.3%)	3182 (63.4%)
**Marital status**			
Single	60 (2.8%)	99 (3.8%)	159 (3.3%)
Married	1929 (90.4%)	2017 (77.6%)	3946 (83.4%)
Others	144 (6.8%)	484 (18.6%)	628 (13.3%)
**Smoking**			
Current smoker	147 (6.5%)	194 (7.1%)	341 (6.8%)
Former smoker	1178 (52.1%)	881 (32.1%)	2059 (41.2%)
Never smoked	936 (41.4%)	1666 (60.8%)	2602 (52.0%)
**Physical activity**			
Inactive	624 (29.0%)	677 (25.9%)	1301 (27.4%)
Moderately inactive	534 (24.9%)	877 (33.6%)	1411 (29.6%)
Moderately active	497 (23.1%)	613 (23.5%)	1110 (23.3%)
Active	493 (23.0%)	447 (17.1%)	940 (19.7%)
**BMI** (kg/m^2) ƚ	26.7 (3.1)	26.0 (4.0)	26.3 (3.6)
**SBP** (mmHg) ƚ	134.9 (16.9)	129.9 (17.3)	132.2 (17.3)
**Alcohol** (units)*	8.0 (3.0-15.0)	3.0 (1.5-8.0)	5.0 (2.0-11.0)
**Pre-existing diseases**			
No	2095 (97.5%)	2499 (98.9%)	4594 (98.2%)
Yes	53 (2.5%)	29 (1.1%)	82 (1.8%)
**Hypnotic use**			
No	2236 (98.6%)	2717 (98.8%)	4953 (98.7%)
Yes	31 (1.4%)	34 (1.2%)	65 (1.3%)
**MDD in the last year**			
No	1964 (97.1%)	2336 (94.3%)	4300 (95.6%)
Yes	58 (2.9%)	142 (5.7%)	200 (4.4%)
**Self-reported general health**			
Excellent	443 (19.7%)	564 (20.6%)	1007 (20.2%)
Good	1549 (68.8%)	1828 (66.8%)	3377 (67.7%)
Moderate	245 (10.9%)	332 (12.1%)	577 (11.6%)
Poor	15 (0.7%)	11 (0.4%)	26 (0.5%)
**Napping**			
No	1309 (61.5%)	1986 (76.9%)	3295 (69.9%)
Yes	819 (38.5%)	598 (23.1%)	1417 (30.1%)
**Time in bed** (h) ƚ	8.5 (0.9)	8.7 (0.8)	8.6 (0.8)
**Sleep duration** (h) ƚ	7.0 (1.1)	6.9 (1.1)	6.9 (1.1)
**CRP** (mg/l)*	1.9 (1.3-3.2)	2.0 (1.3-3.5)	2.0 (1.3-3.4)

Results were presented as ƚ mean (SD), * median (IQR) and the rest N(%). BMI: Body Mass Index; SBP: Systolic Blood Pressure; MDD: Major Depressive Disorder; CRP: C-reactive protein.

For peer review only - http://bmjopen.bmj.com/site/about/guidelines.xhtml

For peer review only

The unadjusted geometric means of CRP were 2.53 (2.44-2.63) mg/l and 2.20 (2.15-2.26) mg/l for those who reported napping and no napping, respectively. Table 2 shows differences in $\log_e$ CRP according to report of napping. After adjustment for age and sex, daytime napping was associated with 10% higher CRP levels [β= 0.10, 95%CI (0.05, 0.15), p<0.001]. With further adjustment for socio-economic status, health-related behaviors, physical health and night time sleep, the association was attenuated but remained borderline significant [β= 0.05, 95%CI (0.00, 0.10), p=0.048]. Table 3 shows the multivariable-adjusted geometric means of CRP. The geometric means of CRP levels were 2.38 (2.29-2.47) mg/l and 2.26 (2.21-2.32) mg/l for those who reported napping and no napping, respectively. When analyses were stratified by sex, the associations between the above napping measure and CRP levels were only significant among women.

The unadjusted geometric means of CRP for those who reported spending <6, 6-8, 8-10 and >10 hours in bed at night were 3.70 (2.48-5.53), 2.25 (2.15-2.35), 2.29 (2.23-2.35) and 2.84 (2.52-3.19) mg/l, respectively. After adjustment for age and sex, spending <6h and >10h in bed were associated with 51% and 17% increase in CRP levels, respectively (table 2). After full adjustments, long time spent in bed was associated with 12% increase in CRP levels while the association between short time in bed and $\log_e$ CRP was not significant. The geometric means of CRP levels among those who spent >10h and <6h in bed were 2.57 (2.30-2.87) and 3.00 (2.04-4.40) mg/l, respectively, higher than those who spent 6-8h in bed [2.28 (2.19-2.38) mg/l]. Night time sleep duration was not associated with $\log_e$ CRP. Repeating the analyses restricted to individuals with CRP levels below 10 mg/L did not change the results.

For peer review only - http://bmjopen.bmj.com/site/about/guidelines.xhtml

Table 2 Linear regression on sleep and log $_e$ CRP in the EPIC-Norfolk cohort study

	All [β (95% CI)]		Men	[β (95% CI)]		Women	[β (95% CI)]	
	Model A	Model B		Model A	Model B		Model A	Model B
**Napping (4712)**			2128			2584		
No (n=3295)	Reference		1309			1986		
Yes (n=1417)	0.10***[0.05,0.15]	0.05*[0.00,0.10]	819	0.08*[0.01,0.15]	0.03[-0.03,0.10]	598	0.12***[0.06,0.19]	0.07*[0.00,0.13]
**Time in bed (4476)**			2036			2440		
<6h (n=13)	0.51*[0.11,0.91]	0.27[-0.11,0.66]	7	0.26[-0.30,0.82]	0.18[-0.37,0.73]	6	0.79**[0.22,1.37]	0.43[-0.12,0.97]
6-8h (n=1101)	Reference		620	Reference		481	Reference	
8-10h (n=3208)	-0.01[-0.06,0.04]	0[-0.05,0.05]	1342	-0.03[-0.10,0.04]	-0.01[-0.09,0.06]	1866	0[-0.07,0.07]	0.02[-0.05,0.09]
>10h (n=154)	0.17**[0.05,0.29]	0.12*[0.00,0.24]	67	0.18[-0.02,0.37]	0.14[-0.05,0.33]	87	0.17*[0.00,0.33]	0.11[-0.05,0.26]
P	0.001	0.09		0.11	0.35		0.01	0.23
**Sleep duration (4795)**			2173			2622		
<6h (n=1232)	0[-0.04,0.05]	-0.02[-0.07,0.02]	476	-0.01[-0.09,0.06]	-0.05[-0.13,0.02]	756	0.02[-0.04,0.08]	-0.01[-0.07,0.05]
6-8h (n=3154)	Reference		1483	Reference		1671	Reference	
>8h (n=409)	0.04[-0.03,0.12]	0[-0.07,0.08]	214	0.08[-0.03,0.19]	0.02[-0.08,0.13]	195	-0.01[-0.11,0.10]	-0.01[-0.11,0.09]
p	0.57	0.59		0.28	0.31		0.86	0.95

Model A: age, sex; Model B: age, sex, social class, education, marital status, BMI, physical activity, smoking, alcohol, self-reported health, pre-existing diseases, SBP, hypnotic use, major depressive disorder, and in women only HRT use. The sleep measures were mutually adjusted in model B. Results were presented as β coefficients (95%CI) of linear regression models using log $_e$ CRP as the outcome.

*p<0.05 ** p<0.01 ***p<0.001

For peer review only - http://bmjopen.bmj.com/site/about/guidelines.xhtml

Table 3 Geometric means (95% CI) of CRP (mg/l) by sleep in the EPIC-Norfolk cohort study

	All		Men			Women		
	Model A	Model B		Model A	Model B		Model A	Model B
**Napping (4712)**			2128			2584		
No (n=3295)	2.23 [2.17, 2.28]***	2.26 [2.21, 2.32]*	1309	2.18 [2.09, 2.27]*	2.22 [2.13, 2.31]	1986	2.27 [2.20, 2.35]***	2.30 [2.23, 2.37]*
Yes (n=1417)	2.47 [2.37, 2.57]	2.38 [2.29, 2.47]	819	2.36 [2.24, 2.49]	2.29 [2.18, 2.41]	598	2.57 [2.43, 2.73]	2.45 [2.31,2.59]
**Time in bed (4476)**			2036			2440		
<6h (n=13)	3.82 [2.56, 5.69]*	3.00 [2.04, 4.40]	7	2.97 [1.70, 5.21]	2.73 [1.58, 4.72]	6	5.09 [2.87, 9.01]**	3.46 [2.01, 5.93]
6-8h (n=1101)	2.30 [2.20, 2.40]	2.28 [2.18, 2.38]	620	2.29 [2.16, 2.43]	2.27 [2.14, 2.41]	481	2.30 [2.16, 2.45]	2.28 [2.14, 2.42]
8-10h (n=3208)	2.27 [2.21, 2.33]	2.29 [2.23, 2.34]	1342	2.22 [2.14,2.32]	2.24 [2.15,2.33]	1866	2.31 [2.23, 2.38]	2.32 [2.25, 2.39]
>10h (n=154)	2.73 [2.43, 3.06]**	2.57 [2.30, 2.87]*	67	2.74 [2.28,3.28]	2.61[2.19,3.11]	87	2.72 [2.34, 3.17]*	2.50 [2.17, 2.89]
**Sleep duration (4795)**			2173			2622		
<6h (n=1232)	2.28 [2.18, 2.37]	2.24 [2.15, 2.33]	476	2.19 [2.05, 2.35]	2.14 [2.00, 2.28]	756	2.34 [2.23, 2.46]	2.30 [2.19, 2.41]
6-8h (n=3154)	2.27 [2.21, 2.33]	2.29 [2.24, 2.35]	1483	2.22 [2.14, 2.31]	2.26 [2.17, 2.34]	1671	2.30 [2.23, 2.38]	2.32 [2.25, 2.40]
>8h (n=409)	2.36 [2.20, 2.53]	2.30 [2.15, 2.46]	214	2.41 [2.18, 2.66]	2.30 [2.09, 2.54]	195	2.29 [2.07, 2.53]	2.29 [2.09, 2.52]

Model A: age, sex; Model B: age, sex, social class, education, marital status, BMI, physical activity, smoking, alcohol, self-reported health, pre-existing diseases, SBP, hypnotic use, major depressive disorder, and in women only HRT use. The sleep measures were mutually adjusted in model B. Results were presented as geometric means (95%CI) of CRP (mg/l).
*p<0.05 ** p<0.01 ***p<0.001; p values were based on comparisons using no napping or 6-8h as the reference category.

For peer review only - http://bmjopen.bmj.com/site/about/guidelines.xhtml

Subgroup analysis investigated differences in these associations by age, sex, social class, smoking, BMI, physical activity and pre-existing diseases (Table 4). These data suggested that the association between napping and CRP levels was stronger for those who were older as compared to those who were younger (p for interaction = 0.007). In addition, the association was more pronounced for extremes of time spent in bed at night (p for interaction=0.01) (Figure 1).

Table 4 The association between $\log_e$ CRP and napping or time spent in bed at night, by subgroups

	**Napping** (Reference: No)		**Time spent in bed at night** (reference: 6-8h)					
	Yes	[β (95% CI)]	0-6h	[β (95% CI)]	8-10h	[β (95% CI)]	>10h	[β (95% CI)]
**Age**	**p for interaction=0.007**		p for interaction=0.66					
<70	0	[-0.06,0.06]	0.14	[-0.25,0.52]	0	[-0.06,0.06]	0.16*	[0.01,0.32]
>70	0.09**	[0.02,0.15]	0.02	[-0.59,0.63]	0.03	[-0.05,0.11]	0.12	[-0.04,0.28]
**Sex**	p for interaction=0.87		p for interaction=0.82					
Men	0.04	[-0.02,0.11]	0.07	[-0.40,0.53]	-0	[-0.08,0.06]	0.16	[-0.03,0.35]
Women	0.05	[-0.01,0.11]	0.15	[-0.33,0.62]	0.02	[-0.04,0.08]	0.1	[-0.04,0.23]
**Social class**	p for interaction=0.38		p for interaction=0.31					
Higher	0.03	[-0.03,0.08]	0.15	[-0.27,0.57]	0.01	[-0.05,0.06]	0.19**	[0.05,0.33]
Lower	0.07	[-0.01,0.14]	0.08	[-0.45,0.61]	0	[-0.08,0.09]	0	[-0.18,0.19]
**Smoking**	p for interaction=0.5		p for interaction=0.55					
Current or former smokers	0.01	[-0.05,0.08]	0.12	[-0.33,0.57]	-0	[-0.10,0.05]	0.12	[-0.04,0.29]
Never smoked	0.06*	[0.01,0.12]	0.11	[-0.37,0.60]	0.03	[-0.03,0.09]	0.11	[-0.04,0.26]
**BMI**	p for interaction=0.73		p for interaction=0.30					
Lower	0.06	[-0.00,0.12]	-0.15	[-0.76,0.46]	0.04	[-0.03,0.10]	0.18*	[0.02,0.34]
Higher	0.07*	[0.01,0.14]	0.36	[-0.05,0.77]	-0	[-0.10,0.04]	0.09	[-0.07,0.25]
**Physical activity**	p for interaction=0.48		p for interaction=0.41					
Inactive	0.06*	[0.00,0.12]	0.28	[-0.14,0.71]	0.02	[-0.04,0.08]	0.13	[-0.00,0.26]
Active	0.02	[-0.05,0.09]	-0.13	[-0.65,0.38]	-0	[-0.10,0.05]	0.08	[-0.14,0.29]
**Pre-existing diseases**	p for interaction=0.05		p for interaction=0.08					
Yes	0.05	[-0.25,0.35]	N/A		0.19	[-0.19,0.56]	0.81**	[0.21,1.41]
No	0.05*	[0.01,0.10]	0.11	[-0.23,0.45]	0.02	[-0.03,0.07]	0.08	[-0.04,0.20]

The model adjusted for age, sex, social class, education, marital status and BMI.
Results were presented as β coefficients of linear regression models using logCRP as the outcome.
*p<0.05 ** p<0.01 ***p<0.001

For peer review only - http://bmjopen.bmj.com/site/about/guidelines.xhtml

Discussion

Our findings from more than 4000 middle- to older-aged English adults suggest that daytime napping was independently associated with higher CRP levels. Those who reported napping had 10% higher CRP levels after adjustment for age and sex. The association was attenuated but remained statistically significant after adjustment for all the covariates. A U-shaped association was observed between time spent in bed at night and CRP levels, though only the effect of long time spent in bed was statistically significant. The napping-CRP associations were more pronounced amongst extremes of time spent in bed at night. No association was found between sleep duration and CRP levels.

To our knowledge, this is the first study to report an association between habitual daytime napping and inflammation. This gives biological insights to the health impact of daytime napping proposed by previous studies.[4, 24] Our study benefits from a large population-based sample, and a wide range of covariates available for adjustment. Although only moderate effect size was detected in the multivariable model, the possibility of over adjustment can not be excluded, given the complexity of the relationship between sleep and health. Overall, this preliminary finding at population level provides an interesting perspective and implies the need for further physiological studies. There are several limitations. Similar to most previous population studies,[24, 25] daytime napping was self-reported as yes or no. It was suggested that power naps (naps< 30min) can be largely beneficial, [2,26]while naps>30min can cause sleep inertia and are not recommended. [29] Without detailed report of napping duration, we were unable to differentiate the effects of power naps from excessive napping. Other sleep disorders (e.g. sleep apnea) that might have led

For peer review only - http://bmjopen.bmj.com/site/about/guidelines.xhtml

For peer review only

to daytime sleepiness were not assessed, so the possibility of residual confounding by these sleep disorders can not be ruled out. Moreover, reported sleep may be a marker of distress levels,[30] which has been linked with CRP levels.[31] However, evaluation of sleep in the primary care setting relies on self-reported data from patients, and also the association remained after adjustment for MDD. Another limitation of this study is that associations are based on a single measurement of CRP. However, the intra-individual variability of CRP measurements is similar to that of other physiologic measures such as blood pressure and cholesterol levels, and a single measurement of CRP has been shown to predict a range of health outcomes.[30,31] Besides, previous study has reported no diurnal variation of CRP concentrations.[34] Notably, while the assessment of napping preceded the CRP measures, baseline CRP level was not measured and there is no information on changes in CRP over time. We were therefore unable to distinguish between napping as a risk factor for, or as an early marker of, increased inflammatory levels. Finally, the current analysis was restricted to the 5018 participants with complete data on CRP and at least one of the sleep measures. Compared to the other participants from the baseline cohort (N=25,639), the present sample were 4 years younger, more likely to be of higher social class and higher education levels. Therefore, our findings have limited generalizability to populations with lower socioeconomic status.

The present study suggested that daytime napping and long time spent in bed at night were associated with higher CRP levels. The observed effect size was similar to previous report on sleep duration and CRP levels, [10] and the increase in CRP levels associated with napping was similar to that associated with 5-year increase in age. [22] While there are no other studies with which to directly compare our findings, the current study is in line with existing evidence on the increased cardiovascular,

For peer review only - http://bmjopen.bmj.com/site/about/guidelines.xhtml

For peer review only

metabolic and mortality risks among those who take naps, found both by other studies and in our study population.[6,7,33,34] Meanwhile, a Greek cohort study suggested that siesta might help to reduce coronary mortality through a stress-releasing mechanism in healthy working men. [37] We also found the association between daytime napping and increased CRP levels only significant among participants over 70 years old when analysis was stratified by age. Daytime napping might have different implications for health among different age groups, and this needs to be considered by future studies. Notably, daytime napping or daytime sleepiness has been associated with the occurrence of degenerative diseases such as Parkinson's disease and dementia in the older population. [38 39] It has also been reported that chronic inflammation, as indicated by an elevation in plasma interleukin (IL)-6 levels, was predictive of future Parkinson's disease.[40] In the current study, daytime napping was associated with a higher serum CRP level, while information on other inflammatory biomarkers was not obtained. Future studies with more complete measures of chronic inflammation might help to clarify the link between daytime napping and degenerative diseases in the elderly. Several studies suggested that the association between sleep and inflammation was only observed in women.[14,18] Consistent with these findings, the association between napping and increased CRP levels was only significant in women, although test for interaction was not statistically significant. The reason for this is unclear, and the gender disparities of sleep physiology need further exploration.

Time spent in bed was rarely studied in relation to CRP concentrations, but long sleep duration has been associated with increased CRP levels.[10,12] Since epidemiology studies have used mixed ways to define sleep duration, the associations between long sleep and inflammation found by some previous studies might actually reflect the effects of long time spent in bed, which was associated with poor general health. [19]

For peer review only - http://bmjopen.bmj.com/site/about/guidelines.xhtml

Indeed, long time spent in bed has been associated with poorer general health, pre-existing health problems, and more frequent use of sleep medications in the EPIC-Norfolk cohort. [19] Therefore, the observed association between long time spent in bed and increased CRP levels might be explained by declining health status. We found no association between sleep duration and CRP levels. This is consistent with findings from the Wisconsin Sleep Cohort Study, which showed no association between CRP and sleep duration, measured both objectively and subjectively.[14] Meanwhile, previous studies have found both short and long sleep duration to be associated with increased CRP levels.[13, 14] Recently, a longitudinal study suggested that each hour per night decrease in sleep duration was associated with 8.1% higher average levels of CRP over a 5-year period. [41] The different correlations with serum CRP levels between the two sleep quantity measures need to be considered by future studies. Interestingly, the association between napping and CRP levels was more pronounced amongst extremes of time in bed. It is possible that extremes of time spent in bed at night partly reflected disturbed night-time sleep, and thereby added to the association for daytime napping. While the complex inter-relationship between night time and daytime sleep is yet to be confirmed, the present study suggested a need for a thorough consideration of both night time and daytime sleep in future studies.

It has been suggested that sleep deprivation is related to the activation of the autonomic nervous system and increased catecholamines, and subsequently stimulate the release of inflammatory mediators. [42]It is unclear biologically why daytime napping was associated with increased levels of inflammatory markers. One possible explanation is through a rise in blood pressure or heart rate upon awakening from a nap, which might be followed by increased endothelial shear stress and the production of inflammatory mediators. [43]Alternatively, daytime napping could be the

For peer review only - http://bmjopen.bmj.com/site/about/guidelines.xhtml

consequence of unnoticed night time sleep disturbance (e.g. sleep apnea) or sleep deprivation, which have been linked with systemic inflammation. [42,44,45] Sleep apnea was not measured in the current study, but the association between daytime napping and serum CRP levels remained after adjustment for BMI and SBP, two strong correlates of sleep apnea.

In the United Kingdom, daytime napping is not part of the cultural norm. It is plausible that those who reported daytime napping and long time spent in bed may represent a population with underlying ill health, and inflammation could be an early sign of these health problems. Although the association between daytime napping and CRP concentrations remained after adjustment for pre-existing diseases, we did find it more pronounced among the older participants, and there might be more existing health problems among this population. Further studies are needed to determine whether daytime napping is a cause for systemic inflammation, or if it is a symptom or consequence of disturbed sleep at night or underlying health problems.

Conclusions

In summary, we found for the first time that daytime napping was associated with increased CRP levels in a population-based middle- to older aged English cohort. The association between daytime napping and CRP levels was more pronounced among older people, and among those who spent extremes of time in bed at night. While the present study provides new insights into the biological effects of daytime napping, causality can not be established from an observational study. Daytime napping might be useful as an independent indicator of people who are at underlying health risk or

For peer review only - http://bmjopen.bmj.com/site/about/guidelines.xhtml

who suffer from disturbed sleep at night. Future physiological studies are needed to gain better understanding of the association.

Acknowledgement

We thank all study participants, general practitioners and the EPIC-Norfolk study team for their contribution.

Competing interests

None

Funding statement: The design and conduct of the EPIC-Norfolk study and collection and management of the data was supported by programme grants from the Medical Research Council UK (G9502233, G0300128) and Cancer Research UK (C865/A2883). YL is supported by the Cambridge Overseas Trust. FPC leads the Sleep Health & Society Programme at the University of Warwick supported, in part, by the University of Warwick RDF and IAS. It has received funding by the NHS National Workforce Projects and the Economic & Social Research Council (ES/K002910/1).

Authors' contributions

The work presented here was carried out in collaboration between all authors. YL and SA analyzed these data and wrote the manuscript with co-authors. YL, SA, NWJW, FPC, PGS, CB and KTK discussed the analysis, interpretation and presentation of these data. RL performed all data management and record linkage. RL and KTK are

For peer review only - http://bmjopen.bmj.com/site/about/guidelines.xhtml

on the management team of EPIC-Norfolk population study and contributed substantially to acquisition of data. KTK is a principal investigator in the EPIC-Norfolk study. All authors provided detailed comments on the draft, and revised the manuscript critically. All authors read and approved the final manuscript.

Data Sharing: No additional data available

For peer review only

For peer review only - http://bmjopen.bmj.com/site/about/guidelines.xhtml

Figure 1: Serum levels of C-reactive protein by napping and time in bed *

* Values are geometric mean CRP (mg/l) across categories adjusted for age, body mass index, physical activity, smoking, alcohol intake, social class, education, marital status, major depressive disorder, self-reported health, pre-existing diseases, systolic blood pressure, hypnotic drug use, and in women only, postmenopausal hormone replacement therapy. The sleep measures were mutually adjusted. Vertical bars represent 95% confidence intervals.

For peer review only

References

1 Milner C, Cote KA. Benefits of napping in healthy adults: impact of nap length, time of day, age, and experience with napping. *J Sleep Res* 2009;**18**:272–81.

2 Bursztyn M, Stessman J. The siesta and mortality: twelve years of prospective observations in 70-year-olds. *Sleep* 2005;**28**:345–7.

3 Jung K-I, Song C-H, Ancoli-Israel S, *et al.* Gender differences in nighttime sleep and daytime napping as predictors of mortality in older adults: the Rancho Bernardo study. *Sleep Med* 2013;**14**:12–9.

4 Tanabe N, Iso H, Seki N, *et al.* Daytime napping and mortality, with a special reference to cardiovascular disease: the JACC study. *Int J Epidemiol* 2010;**39**:233–43.

5 Xu Q, Song Y, Hollenbeck A, *et al.* Day napping and short night sleeping are associated with higher risk of diabetes in older adults. *Diabetes Care* 2010;**33**:78–83.

6 Leng Y, Wainwright NWJ, Cappuccio FP, *et al.* Daytime Napping and the Risk of All-Cause and Cause-Specific Mortality: A 13-Year Follow-up of a British Population. *Am J Epidemiol* 2014;**179**:1115–24.

7 Mullington JM, Simpson NS, Meier-Ewert HK, *et al.* Sleep loss and inflammation. *Best Pract Res Clin Endocrinol Metab* 2010;**24**:775–84.

8 Meier-Ewert HK, Ridker PM, Rifai N, *et al.* Effect of sleep loss on C-reactive protein, an inflammatory marker of cardiovascular risk. *J Am Coll Cardiol* 2004;**43**:678–83.

9 Jackowska M, Kumari M, Steptoe A. Sleep and biomarkers in the English Longitudinal Study of Ageing: associations with C-reactive protein, fibrinogen, dehydroepiandrosterone sulfate and hemoglobin. *Psychoneuroendocrinology* 2013;**38**:1484–93.

For peer review only - http://bmjopen.bmj.com/site/about/guidelines.xhtml

10 Patel SR, Zhu X, Storfer-Isser A, *et al.* Sleep duration and biomarkers of inflammation. *Sleep* 2009;**32**:200–4.

11 Dowd JB, Goldman N, Weinstein M. Sleep duration, sleep quality, and biomarkers of inflammation in a Taiwanese population. *Ann Epidemiol* 2011;**21**:799–806.

12 Grandner MA, Buxton OM, Jackson N, *et al.* Extreme sleep durations and increased C-reactive protein: effects of sex and ethnoracial group. *Sleep* 2013;**36**:769–779E.

13 Miller MA, Kandala N-B, Kivimaki M, *et al.* Gender differences in the cross-sectional relationships between sleep duration and markers of inflammation: Whitehall II study. *Sleep* 2009;**32**:857–64.

14 Taheri S, Austin D, Lin L, *et al.* Correlates of serum C-reactive protein (CRP)--no association with sleep duration or sleep disordered breathing. *Sleep* 2007;**30**:991–6.

15 Faraut B, Boudjeltia KZ, Dyzma M, *et al.* Benefits of napping and an extended duration of recovery sleep on alertness and immune cells after acute sleep restriction. *Brain Behav Immun* 2011;**25**:16–24.

16 Dijk DJ, Duffy JF, Czeisler CA. Contribution of circadian physiology and sleep homeostasis to age-related changes in human sleep. *Chronobiol Int* 2000;**17**:285–311.

17 Foley D, Vitiello M, Bliwise D, *et al.* Frequent napping is associated with excessive daytime sleepiness, depression, pain, and nocturia in older adults. *Am J Geriatr Psychiatry* 2007;**15**:344–50.

18 Ohayon MM, Carskadon M a, Guilleminault C, *et al.* Meta-analysis of quantitative sleep parameters from childhood to old age in healthy individuals: developing normative sleep values across the human lifespan. *Sleep* 2004;**27**:1255–73.

19 Leng Y, Wainwright NWJ, Cappuccio FP, *et al.* Self-reported sleep patterns in a British population cohort. *Sleep Med* 2014;**15**:295–302.

20 Suarez EC. Self-reported symptoms of sleep disturbance and inflammation, coagulation, insulin resistance and psychosocial distress: evidence for gender disparity. *Brain Behav Immun* 2008;**22**:960–8.

21 Day N, Oakes SA, Luben R, *et al.* EPIC-Norfolk: study design and characteristics of the cohort. *Br J Cancer* 1999;**80**:95–103.

22 Ahmadi-Abhari S, Luben RN, Wareham NJ, *et al.* Distribution and determinants of C-reactive protein in the older adult population: European Prospective Investigation into Cancer-Norfolk study. *Eur J Clin Invest* 2013;**43**:899–911.

For peer review only

23 Wareham NJ, Jakes RW, Rennie KL, *et al.* Validity and repeatability of a simple index derived from the short physical activity questionnaire used in the European Prospective Investigation into Cancer and Nutrition (EPIC) study. *Public Health Nutr* 2003;**6**:407–13.

24 Heslop P, Smith GD, Metcalfe C, *et al.* Sleep duration and mortality: The effect of short or long sleep duration on cardiovascular and all-cause mortality in working men and women. *Sleep Med* 2002;**3**:305–14.

25 Hublin C, Partinen M, Koskenvuo M, *et al.* Sleep and mortality: a population-based 22-year follow-up study. *Sleep* 2007;**30**:1245–53.

26 Bursztyn M. The siesta in the elderly: risk factor for mortality? *Arch Intern Med* 1999;**159**:1582–6.

27 Cohen-Mansfield J, Perach R. Sleep duration, nap habits, and mortality in older persons. *Sleep* 2012;**35**:1003–9.

28 Ruggiero JS, Redeker NS. Effects of Napping on Sleepiness and Sleep-Related Performance Deficits in Night-Shift Workers: A Systematic Review. *Biol Res Nurs* 2014;**16**:134–42.

29 Tassi P, Muzet A. Sleep inertia. *Sleep Med Rev* 2000;**4**:341–53.

30 Lavie P. Self-reported sleep duration--what does it mean? *J Sleep Res* 2009;**18**:385–6.

31 Wium-Andersen MK, Ørsted DD, Nielsen SF, *et al.* Elevated C-reactive protein levels, psychological distress, and depression in 73, 131 individuals. *JAMA Psychiatry* 2013;**70**:176–84.

32 Allin KH, Bojesen SE, Nordestgaard BG. Baseline C-reactive protein is associated with incident cancer and survival in patients with cancer. *J Clin Oncol* 2009;**27**:2217–24.

33 Kaptoge S, Di Angelantonio E, Lowe G, *et al.* C-reactive protein concentration and risk of coronary heart disease, stroke, and mortality: an individual participant meta-analysis. *Lancet* 2010;**375**:132–40.

34 Meier-Ewert HK, Ridker PM, Rifai N, *et al.* Absence of diurnal variation of C-reactive protein concentrations in healthy human subjects. *Clin Chem* 2001;**47**:426–30.

35 Campos H, Siles X. Siesta and the risk of coronary heart disease: results from a population-based, case-control study in Costa Rica. *Int J Epidemiol* 2000;**29**:429–37.

36 Lam K-BH, Jiang CQ, Thomas GN, *et al.* Napping is associated with increased risk of type 2 diabetes: the Guangzhou Biobank Cohort Study. *Sleep* 2010;**33**:402–7.

For peer review only - http://bmjopen.bmj.com/site/about/guidelines.xhtml

37 Naska A, Oikonomou E, Trichopoulou A, *et al.* Siesta in healthy adults and coronary mortality in the general population. *Arch Intern Med* 2007;**167**:296–301.

38 Gao J, Huang X, Park Y, *et al.* Daytime napping, nighttime sleeping, and Parkinson disease. *Am J Epidemiol* 2011;**173**:1032–8.

39 Foley D, Monjan A, Masaki K, *et al.* Daytime sleepiness is associated with 3-year incident dementia and cognitive decline in older Japanese-American men. *J Am Geriatr Soc* 2001;**49**:1628–32.

40 Chen H, O'Reilly EJ, Schwarzschild M a, *et al.* Peripheral inflammatory biomarkers and risk of Parkinson's disease. *Am J Epidemiol* 2008;**167**:90–5.

41 Ferrie JE, Kivimäki M, Akbaraly TN, *et al.* Associations Between Change in Sleep Duration and Inflammation: Findings on C-reactive Protein and Interleukin 6 in the Whitehall II Study. *Am J Epidemiol* 2013;**178**:956–61.

42 Mullington JM, Haack M, Toth M, *et al.* Cardiovascular, inflammatory, and metabolic consequences of sleep deprivation. *Prog Cardiovasc Dis* 2009;**51**:294–302.

43 Chiu J-J, Usami S, Chien S. Vascular endothelial responses to altered shear stress: pathologic implications for atherosclerosis. *Ann Med* 2009;**41**:19–28.

44 Liukkonen T, Räsänen P, Ruokonen A, *et al.* C-reactive protein levels and sleep disturbances: observations based on the Northern Finland 1966 Birth Cohort study. *Psychosom Med* 2007;**69**:756–61.

45 Svensson M, Venge P, Janson C, *et al.* Relationship between sleep-disordered breathing and markers of systemic inflammation in women from the general population. *J Sleep Res* 2012;**21**:147–54.

For peer review only

For peer review only - http://bmjopen.bmj.com/site/about/guidelines.xhtml

For peer review only

For peer review only - http://bmjopen.bmj.com/site/about/guidelines.xhtml

Daytime napping, sleep duration and serum C-reactive protein: a population-based cohort study

Yue Leng, M,Phil[1*], Sara Ahmadi-Abhari, MD[1], Nick W.J. Wainwright, PhD[1], Francesco P. Cappuccio, FRCP, FAHA[2], Paul G. Surtees, PhD[1], Robert Luben, MSc[1], Carol Brayne, MD, FRCP [3], Kay-Tee Khaw, MD, FRCP[1]

Affiliations
[1] Department of Public Health and Primary Care, Strangeways Research Laboratory, University of Cambridge, Cambridge, UK
[2] Division of Mental Health & Wellbeing, Warwick Medical School, University of Warwick, Coventry, UK
[3] Department of Public Health and Primary Care, Institute of Public Health, University of Cambridge, Cambridge, UK

Word count: 3195 (Main text, excluding title page, abstract, references, figures and tables);

[*] Address for correspondence: Yue Leng, Department of Public Health and Primary Care, Strangeways Research Laboratory, University of Cambridge, Cambridge CB1 8RN, UK (Email: yl411@medschl.cam.ac.uk; Tel: +44(0)1223748686)

For peer review only - http://bmjopen.bmj.com/site/about/guidelines.xhtml

ABSTRACT

Objectives: To explore whether daytime napping and sleep duration are linked to serum C-reactive protein (CRP), a pro-inflammatory marker, in an older-aged British population.

Design: Cross-sectional ~~Population-based prospective cohort~~ study

Setting: European Prospective Investigation into Cancer and Nutrition (EPIC)-Norfolk study.

Participants: A total of 5018 men and women aged 48-92 years reported their sleep habits and had serum CRP levels measured.

Outcome and measures: CRP was measured (mg/l) during 2006-2011 in fresh blood samples using high-sensitivity methods. Participants reported napping habits during 2002-2004, and reported sleep quantity during 2006-2007. Multivariable linear regression models were used to examine the association between napping and log-transformed CRP, and geometric mean CRP levels were calculated.

Results: After adjustment for age and sex, those who reported napping had 10% higher CRP levels compared to those not napping. The association was attenuated but remained borderline significant [β= 0.05 (95% CI: 0.00, 0.10)] after further adjustment for social class, education, marital status, body mass index, physical activity, smoking, alcohol intake, self-reported health, pre-existing diseases, systolic blood pressure, hypnotic drug use, depression and in women only hormone replacement therapy use. The geometric means (95%CI) of CRP levels were 2.38 (2.29-2.47) mg/l and 2.26 (2.21-2.32) mg/l for those who reported napping and no

For peer review only - http://bmjopen.bmj.com/site/about/guidelines.xhtml

For peer review only

napping, respectively. A U-shaped association was observed between time spent in bed at night and CRP levels, and night time sleep duration was not associated with serum CRP levels. The association between napping and CRP was stronger for older participants, and amongst extremes of time spent in bed at night.

Conclusions: Daytime napping was associated with increased CRP levels in an older-aged British population. Further studies are needed to determine whether daytime napping is a cause for systemic inflammation, or if it is a symptom or consequence of underlying health problems.

Keywords: *Sleep; Napping; C-reactive protein; Inflammation; Population*

For peer review only

Article summary

Article focus:

- Daytime napping has been associated with cardiovascular disease and mortality, but the biological implication of napping is unclear.
- To our knowledge, this is the first population-based study to examine the association between daytime napping and serum CRP levels.
- We also studied both time spent in bed at night and sleep duration in relation to CRP levels.

Key messages:

- Daytime napping was associated with 10% higher CRP levels, independent of age and sex.
- The association was more pronounced in women, older participants, and amongst extremes of time spent in bed at night.
- Sleep duration was not associated with CRP levels.

Strengths and limitations:

- Our study benefits from a large population-based sample, measures of both daytime and night time sleep, and a wide range of covariates available for adjustment.
- Sleep habits were self-reported
- The purpose and duration of napping was not reported, and sleep apnea was not measured.

For peer review only - http://bmjopen.bmj.com/site/about/guidelines.xhtml

Introduction

Recently there has been growing interest in studying the health implications of daytime napping. Although the benefits of napping on wakefulness performance have been widely documented, especially among shift workers, [1] a few studies also reported an increased health risk associated with daytime napping. Specifically, daytime napping has been associated with increased mortality, [2,3] cardiovascular, [4]and diabetes risk. [5] We have recently found a 32% increased mortality risk among those who napped for ≥1h/ day in a middle- to older- aged English population. [6] While it is not yet clear whether daytime napping is a cause or symptom of the increased health risk, examination of the physiological correlations of napping will help to gain understanding of the napping-health relationship.

One important proposed pathway for the association between sleep and health outcomes is through inflammation.[7] C-reative protein (CRP), a general marker for inflammation, was first suggested to increase after sleep depreviation in an experimental study.[8] Since then, there have been increasing numbers of observational studies which produced mixed findings on the association. Long sleep duration has been associated with increasing CRP among men,[9] women,[10] as well as in the sexes combined sample,[11,12] with long sleep being brought up as a potential marker of underlying inflammatory illness. [11] By contrast, Miller et al [13] found an association between short sleep duration and increased CRP only in women. Taheri et al suggested no significant association between CRP levels and sleep duration in the Wisconsin Sleep Cohort Study.[14]

Despite the growing interest into the inflammatory correlates of night time sleep, there has been limited evidence on the physiological effects of daytime napping.

For peer review only - http://bmjopen.bmj.com/site/about/guidelines.xhtml

Experimental study has reported a beneficial effect of napping on immune cells following acute sleep restriction.[15] However, habitual daytime napping as a lifestyle might relate to different physiological effects compared to napping as a form of recovery sleep, and understanding the link between habitual napping and inflammation could help to clarify the association observed for napping and the increased mortality risk. Moreover, a number of changes in sleep from middle to older adulthood have been reported, including an advanced circadian pacemaker, decreased sleep efficiency and increased daytime sleepiness. [16,17,18] Therefore we set out to examine the associations between daytime napping and serum CRP levels in the European Prospective Investigation into Cancer (EPIC)-Norfolk cohort ~~study~~, a middle- to older- aged British population . In addition, we have previously reported the different implications between sleep duration and time spent in bed, [19] so confirmatory analysis was also conducted to test the associations separately for sleep duration and time spent in bed at night. Given the gender disparity suggested by some previous studies,[14, 18] we explored sex differences in the associations.

Methods

Participants

The design and study methods of EPIC-Norfolk have been described previously.[21]Briefly, 25,639 men and women were recruited into the EPIC-Norfolk study during 1993-1997 using general practice age-sex registers, and attended the baseline health check. These participants were then followed up for two further health examinations from 1996 to 2000 and from 2006 to 2011. In between these health examinations, participants were sent questionnaires for completion and returned by

For peer review only - http://bmjopen.bmj.com/site/about/guidelines.xhtml

For peer review only

post. The Norwich District Ethics Committee approved the study and all participants gave signed informed consent.

Sleep Measures

Habitual daytime napping was ascertained during 2002-2004, by asking participants the question “Do you normally take a nap during the day?”.

“Night time sleep duration” and “Time spent in bed at night” were ascertained during 2006-2007, by asking participants “On average, about how many hours have you slept each night?”; “At what time do you normally get up?” and “At what time do you normally go to bed?”. “Time spent in bed” was derived from the differences between rise time and bedtime, and as a weighted mean measure of weekday and weekend times [5/7*(time on a week- day)+ 2/7*(time on a weekend day)].

CRP

CRP was measured (mg/l) at the third health examination (2006-2011) in fresh blood samples using high-sensitivity methods using the Siemens Dimension clinical chemistry analyzer (Siemens Dimension clinical chemistry analyzer, Newark, Delaware, US), with between batch CV values of 3.1% at 7.86 mg/l, 3.7% at 88.75 mg/l.

Covariates

Covariates measured closer in time to each measure of sleep were chosen and included in the models for the corresponding sleep measures. The covariates were chosen as a priori based on their link with CRP and sleep.[19,22] Social class (professionals, managerial and technical occupations, skilled workers subdivided into

For peer review only - http://bmjopen.bmj.com/site/about/guidelines.xhtml

non-manual and manual, partly skilled workers and unskilled manual workers) and education (highest qualification attained: no qualifications, educated to age 16, educated to age 18, and educated to degree level) were assessed at the baseline questionnaire. Body Mass Index (BMI; weight in kilograms divided by height in meters squared) and Systolic Blood Pressure (SBP) (mmHg) were objectively assessed through the health examination. The other covariates reported by questionnaires included: marital status (single, married, widowed, separated and divorced), physical activity (inactive, moderately inactive, moderately active and active[23]), smoking status(current, former and non-smokers), alcohol intake (units of alcohol drunk per week), self-reported general health (excellent, good, moderate or poor), Major Depressive Disorder (MDD) (Yes/No), pre-existing diseases (including self-reported stroke, myocardial infarction, diabetes, cancer, asthma, bronchitis, and emphysema), use of hypnotic drugs (Yes/No), and Hormone Replacement Therapy (HRT) use.

Statistical analysis

Analysis was restricted to those participants with complete data on all covariates. A total of 5018 participants had complete data on CRP and at least one of the sleep measures and were included in the analysis.

The CRP values were natural-log transformed ($\log_e$ CRP) to approximate a normal distribution. Due to the U-shaped relationship reported by previous studies on sleep duration and health risk,[22,23] and in order to retain sufficient numbers, sleep duration was categorized into three categories (<6, 6-8, and >8 hours) and time spent in bed was categorized into four categories (<6, 6-8, 8-10, and >10 hours). Multivariable linear regression models using $\log_e$ CRP as the outcome were conducted to examine

For peer review only - http://bmjopen.bmj.com/site/about/guidelines.xhtml

the associations between each sleep measure (daytime napping [N=4712], time spent in bed [N=4476] and sleep duration [N=4795]) and CRP levels. For each sleep variable, results were presented: A. adjusted for age, sex; B. further adjusted for social class, education and marital status, BMI, physical activity, smoking and alcohol intake, depression, self-reported health, pre-existing diseases, SBP, hypnotic drug use and in women only postmenopausal HRT. The sleep measures were mutually adjusted in model B. Since the associations for sleep duration and time in bed might be non-linear (approximately U-shaped), the β coefficients (with 95% confidence intervals) were calculated with 6-8h being the reference groups for the examination of sleep quantitative measures. For analysis by napping habit, the no napping category was chosen as reference group. Results were presented for the whole sample and for men and women separately.

Geometric-mean (95% confidence interval, CI) serum CRP values (mg/l) were calculated by exponentiating crude and adjusted-least square mean (95% CI) $\log_e$ CRP values. Finally, subgroup analysis was conducted for the main sleep variable of interest, napping and time spent in bed at night, to explore the effects of potential effect modifiers (including age, sex, social class, smoking, BMI, physical activity and pre-existing diseases). These analyses were adjusted for age, sex, social class, education, marital status and BMI. The two by two interactions between napping habit, time in bed, and sleep duration were examined using the likelihood ratio test. Sensitivity analysis All statistical tests were two-sided, and a p value <0.05 was considered statistically significant. Analyses were implemented in STATA 12.0.

For peer review only

Results

Characteristics of the study sample (2267 men, 2751 women) are shown in table 1. The mean age of the participants was 69.5 (ranging from 48 to 92) years old. More than 60% of the sample came from non-manual social class, and 48.0% were former or current smokers. A total of 38.5% men and 23.1% women reported daytime napping. The average time people spent in bed every night was 8.6±0.8 h and the average sleep duration was 6.9±1.1 h. Serum CRP ranged from 0.1-116.6 mg/l, with a median of 2.0 mg/l (interquartile range 2.07 mg/l).

For peer review only

Table 1 Baseline characteristics of the EPIC-Norfolk participants

	Men	Women	Total
**Age** (years) ƚ	70.5 (8.0)	68.6 (7.9)	69.5 (8.0)
**Social class**			
Non-manual	1495 (66.5%)	1885 (69.3%)	3380 (68.0%)
Manual	753 (33.5%)	836 (30.7%)	1589 (32.0%)
**Education**			
Lower	686 (30.3%)	1148 (41.7%)	1834 (36.6%)
Higher	1580 (69.7%)	1602 (58.3%)	3182 (63.4%)
**Marital status**			
Single	60 (2.8%)	99 (3.8%)	159 (3.3%)
Married	1929 (90.4%)	2017 (77.6%)	3946 (83.4%)
Others	144 (6.8%)	484 (18.6%)	628 (13.3%)
**Smoking**			
Current smoker	147 (6.5%)	194 (7.1%)	341 (6.8%)
Former smoker	1178 (52.1%)	881 (32.1%)	2059 (41.2%)
Never smoked	936 (41.4%)	1666 (60.8%)	2602 (52.0%)
**Physical activity**			
Inactive	624 (29.0%)	677 (25.9%)	1301 (27.4%)
Moderately inactive	534 (24.9%)	877 (33.6%)	1411 (29.6%)
Moderately active	497 (23.1%)	613 (23.5%)	1110 (23.3%)
Active	493 (23.0%)	447 (17.1%)	940 (19.7%)
**BMI** (kg/m^2) ƚ	26.7 (3.1)	26.0 (4.0)	26.3 (3.6)
**SBP** (mmHg) ƚ	134.9 (16.9)	129.9 (17.3)	132.2 (17.3)
**Alcohol** (units)*	8.0 (3.0-15.0)	3.0 (1.5-8.0)	5.0 (2.0-11.0)
**Pre-existing diseases**			
No	2095 (97.5%)	2499 (98.9%)	4594 (98.2%)
Yes	53 (2.5%)	29 (1.1%)	82 (1.8%)
**Hypnotic use**			
No	2236 (98.6%)	2717 (98.8%)	4953 (98.7%)
Yes	31 (1.4%)	34 (1.2%)	65 (1.3%)
**MDD in the last year**			
No	1964 (97.1%)	2336 (94.3%)	4300 (95.6%)
Yes	58 (2.9%)	142 (5.7%)	200 (4.4%)
**Self-reported general health**			
Excellent	443 (19.7%)	564 (20.6%)	1007 (20.2%)
Good	1549 (68.8%)	1828 (66.8%)	3377 (67.7%)
Moderate	245 (10.9%)	332 (12.1%)	577 (11.6%)
Poor	15 (0.7%)	11 (0.4%)	26 (0.5%)
**Napping**			
No	1309 (61.5%)	1986 (76.9%)	3295 (69.9%)
Yes	819 (38.5%)	598 (23.1%)	1417 (30.1%)
**Time in bed** (h) ƚ	8.5 (0.9)	8.7 (0.8)	8.6 (0.8)
**Sleep duration** (h) ƚ	7.0 (1.1)	6.9 (1.1)	6.9 (1.1)
**CRP** (mg/l)*	1.9 (1.3-3.2)	2.0 (1.3-3.5)	2.0 (1.3-3.4)

Results were presented as ƚ mean (SD), * median (IQR) and the rest N(%). BMI: Body Mass Index; SBP: Systolic Blood Pressure; MDD: Major Depressive Disorder; CRP: C-reactive protein.

For peer review only - http://bmjopen.bmj.com/site/about/guidelines.xhtml

The unadjusted geometric means of CRP were 2.53 (2.44-2.63) mg/l and 2.20 (2.15-2.26) mg/l for those who reported napping and no napping, respectively. Table 2 shows differences in $\log_e$ CRP according to report of napping. After adjustment for age and sex, daytime napping was associated with 10% higher CRP levels [β= 0.10, 95%CI (0.05, 0.15), p<0.001]. With further adjustment for socio-economic status, health-related behaviors, physical health and night time sleep, the association was attenuated but remained borderline significant [β= 0.05, 95%CI (0.00, 0.10), p=0.048]. Table 3 shows the multivariable-adjusted geometric means of CRP. The geometric means of CRP levels were 2.38 (2.29-2.47) mg/l and 2.26 (2.21-2.32) mg/l for those who reported napping and no napping, respectively. When analyses were stratified by sex, the associations between the above napping measure and CRP levels were only significant among women.

The unadjusted geometric means of CRP for those who reported spending <6, 6-8, 8-10 and >10 hours in bed at night were 3.70 (2.48-5.53), 2.25 (2.15-2.35), 2.29 (2.23-2.35) and 2.84 (2.52-3.19) mg/l, respectively. After adjustment for age and sex, spending <6h and >10h in bed were associated with 51% and 17% increase in CRP levels, respectively (table 2). After full adjustments, long time spent in bed was associated with 12% increase in CRP levels while the association between short time in bed and $\log_e$ CRP was not significant. The geometric means of CRP levels among those who spent >10h and <6h in bed were 2.57 (2.30-2.87) and 3.00 (2.04-4.40) mg/l, respectively, higher than those who spent 6-8h in bed [2.28 (2.19-2.38) mg/l]. Night time sleep duration was not associated with $\log_e$ CRP. Repeating the analyses restricted to individuals with CRP levels below 10 mg/L did not change the results.

For peer review only - http://bmjopen.bmj.com/site/about/guidelines.xhtml

Table 2 Linear regression on sleep and log $_e$ CRP in the EPIC-Norfolk cohort study

	All [β (95% CI)]		Men	[β (95% CI)]		Women	[β (95% CI)]	
	Model A	Model B		Model A	Model B		Model A	Model B
**Napping (4712)**			2128			2584		
No (n=3295)	Reference		1309			1986		
Yes (n=1417)	0.10***[0.05,0.15]	0.05*[0.00,0.10]	819	0.08*[0.01,0.15]	0.03[-0.03,0.10]	598	0.12***[0.06,0.19]	0.07*[0.00,0.13]
**Time in bed (4476)**			2036			2440		
<6h (n=13)	0.51*[0.11,0.91]	0.27[-0.11,0.66]	7	0.26[-0.30,0.82]	0.18[-0.37,0.73]	6	0.79**[0.22,1.37]	0.43[-0.12,0.97]
6-8h (n=1101)	Reference		620	Reference		481	Reference	
8-10h (n=3208)	-0.01[-0.06,0.04]	0[-0.05,0.05]	1342	-0.03[-0.10,0.04]	-0.01[-0.09,0.06]	1866	0[-0.07,0.07]	0.02[-0.05,0.09]
>10h (n=154)	0.17**[0.05,0.29]	0.12*[0.00,0.24]	67	0.18[-0.02,0.37]	0.14[-0.05,0.33]	87	0.17*[0.00,0.33]	0.11[-0.05,0.26]
P	0.001	0.09		0.11	0.35		0.01	0.23
**Sleep duration (4795)**			2173			2622		
<6h (n=1232)	0[-0.04,0.05]	-0.02[-0.07,0.02]	476	-0.01[-0.09,0.06]	-0.05[-0.13,0.02]	756	0.02[-0.04,0.08]	-0.01[-0.07,0.05]
6-8h (n=3154)	Reference		1483	Reference		1671	Reference	
>8h (n=409)	0.04[-0.03,0.12]	0[-0.07,0.08]	214	0.08[-0.03,0.19]	0.02[-0.08,0.13]	195	-0.01[-0.11,0.10]	-0.01[-0.11,0.09]
p	0.57	0.59		0.28	0.31		0.86	0.95

Model A: age, sex; Model B: age, sex, social class, education, marital status, BMI, physical activity, smoking, alcohol, self-reported health, pre-existing diseases, SBP, hypnotic use, major depressive disorder, and in women only HRT use. The sleep measures were mutually adjusted in model B. Results were presented as β coefficients (95%CI) of linear regression models using log $_e$ CRP as the outcome.
*p<0.05 ** p<0.01 ***p<0.001

For peer review only - http://bmjopen.bmj.com/site/about/guidelines.xhtml

Table 3 Geometric means (95% CI) of CRP (mg/l) by sleep in the EPIC-Norfolk cohort study

	All		Men			Women		
	Model A	Model B		Model A	Model B		Model A	Model B
**Napping (4712)**			2128			2584		
No (n=3295)	2.23 [2.17, 2.28]***	2.26 [2.21, 2.32]*	1309	2.18 [2.09, 2.27]*	2.22 [2.13, 2.31]	1986	2.27 [2.20, 2.35]***	2.30 [2.23, 2.37]*
Yes (n=1417)	2.47 [2.37, 2.57]	2.38 [2.29, 2.47]	819	2.36 [2.24, 2.49]	2.29 [2.18, 2.41]	598	2.57 [2.43, 2.73]	2.45 [2.31,2.59]
**Time in bed (4476)**			2036			2440		
<6h (n=13)	3.82 [2.56, 5.69]*	3.00 [2.04, 4.40]	7	2.97 [1.70, 5.21]	2.73 [1.58, 4.72]	6	5.09 [2.87, 9.01]**	3.46 [2.01, 5.93]
6-8h (n=1101)	2.30 [2.20, 2.40]	2.28 [2.18, 2.38]	620	2.29 [2.16, 2.43]	2.27 [2.14, 2.41]	481	2.30 [2.16, 2.45]	2.28 [2.14, 2.42]
8-10h (n=3208)	2.27 [2.21, 2.33]	2.29 [2.23, 2.34]	1342	2.22 [2.14,2.32]	2.24 [2.15,2.33]	1866	2.31 [2.23, 2.38]	2.32 [2.25, 2.39]
>10h (n=154)	2.73 [2.43, 3.06]**	2.57 [2.30, 2.87]*	67	2.74 [2.28,3.28]	2.61[2.19,3.11]	87	2.72 [2.34, 3.17]*	2.50 [2.17, 2.89]
**Sleep duration (4795)**			2173			2622		
<6h (n=1232)	2.28 [2.18, 2.37]	2.24 [2.15, 2.33]	476	2.19 [2.05, 2.35]	2.14 [2.00, 2.28]	756	2.34 [2.23, 2.46]	2.30 [2.19, 2.41]
6-8h (n=3154)	2.27 [2.21, 2.33]	2.29 [2.24, 2.35]	1483	2.22 [2.14, 2.31]	2.26 [2.17, 2.34]	1671	2.30 [2.23, 2.38]	2.32 [2.25, 2.40]
>8h (n=409)	2.36 [2.20, 2.53]	2.30 [2.15, 2.46]	214	2.41 [2.18, 2.66]	2.30 [2.09, 2.54]	195	2.29 [2.07, 2.53]	2.29 [2.09, 2.52]

Model A: age, sex; Model B: age, sex, social class, education, marital status, BMI, physical activity, smoking, alcohol, self-reported health, pre-existing diseases, SBP, hypnotic use, major depressive disorder, and in women only HRT use. The sleep measures were mutually adjusted in model B. Results were presented as geometric means (95%CI) of CRP (mg/l).

*p<0.05 ** p<0.01 ***p<0.001; p values were based on comparisons using no napping or 6-8h as the reference category.

For peer review only

For peer review only - http://bmjopen.bmj.com/site/about/guidelines.xhtml

Subgroup analysis investigated differences in these associations by age, sex, social class, smoking, BMI, physical activity and pre-existing diseases (Table 4). These data suggested that the association between napping and CRP levels was stronger for those who were older as compared to those who were younger (p for interaction = 0.007). In addition, the association was more pronounced for extremes of time spent in bed at night (p for interaction=0.01) (Figure 1).

Table 4 The association between log $_e$ CRP and napping or time spent in bed at night, by subgroups

	**Napping** (Reference: No)		**Time spent in bed at night** (reference: 6-8h)					
	Yes	[β (95% CI)]	0-6h	[β (95% CI)]	8-10h	[β (95% CI)]	>10h	[β (95% CI)]
**Age**	**p for interaction=0.007**		p for interaction=0.66					
<70	0	[-0.06,0.06]	0.14	[-0.25,0.52]	0	[-0.06,0.06]	0.16*	[0.01,0.32]
>70	0.09**	[0.02,0.15]	0.02	[-0.59,0.63]	0.03	[-0.05,0.11]	0.12	[-0.04,0.28]
**Sex**	p for interaction=0.87		p for interaction=0.82					
Men	0.04	[-0.02,0.11]	0.07	[-0.40,0.53]	-0	[-0.08,0.06]	0.16	[-0.03,0.35]
Women	0.05	[-0.01,0.11]	0.15	[-0.33,0.62]	0.02	[-0.04,0.08]	0.1	[-0.04,0.23]
**Social class**	p for interaction=0.38		p for interaction=0.31					
Higher	0.03	[-0.03,0.08]	0.15	[-0.27,0.57]	0.01	[-0.05,0.06]	0.19**	[0.05,0.33]
Lower	0.07	[-0.01,0.14]	0.08	[-0.45,0.61]	0	[-0.08,0.09]	0	[-0.18,0.19]
**Smoking**	p for interaction=0.5		p for interaction=0.55					
Current or former smokers	0.01	[-0.05,0.08]	0.12	[-0.33,0.57]	-0	[-0.10,0.05]	0.12	[-0.04,0.29]
Never smoked	0.06*	[0.01,0.12]	0.11	[-0.37,0.60]	0.03	[-0.03,0.09]	0.11	[-0.04,0.26]
**BMI**	p for interaction=0.73		p for interaction=0.30					
Lower	0.06	[-0.00,0.12]	-0.15	[-0.76,0.46]	0.04	[-0.03,0.10]	0.18*	[0.02,0.34]
Higher	0.07*	[0.01,0.14]	0.36	[-0.05,0.77]	-0	[-0.10,0.04]	0.09	[-0.07,0.25]
**Physical activity**	p for interaction=0.48		p for interaction=0.41					
Inactive	0.06*	[0.00,0.12]	0.28	[-0.14,0.71]	0.02	[-0.04,0.08]	0.13	[-0.00,0.26]
Active	0.02	[-0.05,0.09]	-0.13	[-0.65,0.38]	-0	[-0.10,0.05]	0.08	[-0.14,0.29]
**Pre-existing diseases**	p for interaction=0.05		p for interaction=0.08					
Yes	0.05	[-0.25,0.35]	N/A		0.19	[-0.19,0.56]	0.81**	[0.21,1.41]
No	0.05*	[0.01,0.10]	0.11	[-0.23,0.45]	0.02	[-0.03,0.07]	0.08	[-0.04,0.20]

The model adjusted for age, sex, social class, education, marital status and BMI.
Results were presented as β coefficients of linear regression models using logCRP as the outcome.
*p<0.05 ** p<0.01 ***p<0.001

For peer review only - http://bmjopen.bmj.com/site/about/guidelines.xhtml

Discussion

Our findings from more than 4000 middle- to older-aged English adults suggest that daytime napping was independently associated with higher CRP levels. Those who reported napping had 10% higher CRP levels after adjustment for age and sex. The association was attenuated but remained statistically significant after adjustment for all the covariates. A U-shaped association was observed between time spent in bed at night and CRP levels, though only the effect of long time spent in bed was statistically significant. The napping-CRP associations were more pronounced amongst extremes of time spent in bed at night. No association was found between sleep duration and CRP levels.

To our knowledge, this is the first study to report an association between habitual daytime napping and inflammation. This gives biological insights to the health impact of daytime napping proposed by previous studies.[4, 24] Our study benefits from a large population-based sample, and a wide range of covariates available for adjustment. Although only moderate effect size was detected in the multivariable model, the possibility of over adjustment can not be excluded, given the complexity of the relationship between sleep and health. Overall, -this preliminary finding at population level provides an interesting perspective and implies the need for further physiological studies. There are several limitations. Similar to most previous population studies,[24, 25] daytime napping was self-reported as yes or no. It was suggested that power naps (naps< 30min) can be largely beneficial, [2,26]while naps>30min can cause sleep inertia and are not recommended. [29] Without detailed report of napping duration, we were unable to differentiate the effects of power naps from excessive napping. Other sleep disorders (e.g. sleep apnea) that might have led

For peer review only - http://bmjopen.bmj.com/site/about/guidelines.xhtml

to daytime sleepiness were not assessed, so the possibility of residual confounding by these sleep disorders can not be ruled out. Moreover, reported sleep may be a marker of distress levels,[30] which has been linked with CRP levels.[31] However, evaluation of sleep in the primary care setting relies on self-reported data from patients, and also the association remained after adjustment for MDD. Another limitation of this study is that associations are based on a single measurement of CRP. However, the intra-individual variability of CRP measurements is similar to that of other physiologic measures such as blood pressure and cholesterol levels, and a single measurement of CRP has been shown to predict a range of health outcomes.[30,31] Besides, previous study has reported no diurnal variation of CRP concentrations.[34] Notably, while the assessment of napping preceded the CRP measures, baseline CRP level was not measured and there is no information on changes in CRP over time. We were therefore unable to distinguish between napping as a risk factor for, or as an early marker of, increased inflammatory levels. Finally, the current analysis was restricted to the 5018 participants with complete data on CRP and at least one of the sleep measures. Compared to the other participants from the baseline cohort (N=25,639), the present sample were 4 years younger, more likely to be of higher social class and higher education levels. Therefore, our findings have limited generalizability to populations with lower socioeconomic status.

The present study suggested that daytime napping and long time spent in bed at night were associated with higher CRP levels. The observed effect size was similar to previous report on sleep duration and CRP levels, [10] and the increase in CRP levels associated with napping was similar to that associated with 5-year increase in age. [22] While there are no other studies with which to directly compare our findings, the current study is in line with existing evidence on the increased cardiovascular,

For peer review only - http://bmjopen.bmj.com/site/about/guidelines.xhtml

metabolic and mortality risks among those who take naps, found both by other studies and in our study population.[6,7,33,34] Meanwhile, a Greek cohort study suggested that siesta might help to reduce coronary mortality through a stress-releasing mechanism in healthy working men. [37] We also found the association between daytime napping and increased CRP levels only significant among participants over 70 years old when analysis was stratified by age. Daytime napping might have different implications for health among different age groups, and this needs to be considered by future studies. Notably, daytime napping or daytime sleepiness has been associated with the occurrence of degenerative diseases such as Parkinson's disease and dementia in the older population. [38 39] It has also been reported that chronic inflammation, as indicated by an elevation in plasma interleukin (IL)-6 levels, was predictive of future Parkinson's disease.[40] In the current study, daytime napping was associated with a higher serum CRP level, while information on other inflammatory biomarkers was not obtained. Future studies with more complete measures of chronic inflammation might help to clarify the link between daytime napping and degenerative diseases in the elderly. Several studies suggested that the association between sleep and inflammation was only observed in women.[14,18] Consistent with these findings, the association between napping and increased CRP levels was only significant in women, although test for interaction was not statistically significant. The reason for this is unclear, and the gender disparities of sleep physiology need further exploration.

Time spent in bed was rarely studied in relation to CRP concentrations, but long sleep duration has been associated with increased CRP levels.[10,12] Since epidemiology studies have used mixed ways to define sleep duration, the associations between long sleep and inflammation found by some previous studies might actually reflect the effects of long time spent in bed, which was associated with poor general health. [19]

For peer review only - http://bmjopen.bmj.com/site/about/guidelines.xhtml

Indeed, long time spent in bed has been associated with poorer general health, pre-existing health problems, and more frequent use of sleep medications in the EPIC-Norfolk cohort. [19] Therefore, the observed association between long time spent in bed and increased CRP levels might be explained by declining health status. We found no association between sleep duration and CRP levels. This is consistent with findings from the Wisconsin Sleep Cohort Study, which showed no association between CRP and sleep duration, measured both objectively and subjectively.[14] Meanwhile, previous studies have found both short and long sleep duration to be associated with increased CRP levels.[13, 14] Recently, a longitudinal study suggested that each hour per night decrease in sleep duration was associated with 8.1% higher average levels of CRP over a 5-year period. [41] The different correlations with serum CRP levels between the two sleep quantity measures need to be considered by future studies. Interestingly, the association between napping and CRP levels was more pronounced amongst extremes of time in bed. It is possible that extremes of time spent in bed at night partly reflected disturbed night-time sleep, and thereby added to the association for daytime napping. While the complex inter-relationship between night time and daytime sleep is yet to be confirmed, the present study suggested a need for a thorough consideration of both night time and daytime sleep in future studies.

It has been suggested that sleep deprivation is related to the activation of the autonomic nervous system and increased catecholamines, and subsequently stimulate the release of inflammatory mediators. [42]It is unclear biologically why daytime napping was associated with increased levels of inflammatory markers. One possible explanation is through a rise in blood pressure or heart rate upon awakening from a nap, which might be followed by increased endothelial shear stress and the production of inflammatory mediators. [43]Alternatively, daytime napping could be the

For peer review only - http://bmjopen.bmj.com/site/about/guidelines.xhtml

consequence of unnoticed night time sleep disturbance (e.g. sleep apnea) or sleep deprivation, which have been linked with systemic inflammation. [42,44,45] Sleep apnea was not measured in the current study, but the association between daytime napping and serum CRP levels remained after adjustment for BMI and SBP, two strong correlates of sleep apnea.

In the United Kingdom, daytime napping is not part of the cultural norm. It is plausible that those who reported daytime napping and long time spent in bed may represent a population with underlying ill health, and inflammation could be an early sign of these health problems. Although the association between daytime napping and CRP concentrations remained after adjustment for pre-existing diseases, we did find it more pronounced among the older participants, and there might be more existing health problems among this population. Further studies are needed to determine whether daytime napping is a cause for systemic inflammation, or if it is a symptom or consequence of disturbed sleep at night or underlying health problems.

Conclusions

In summary, we found for the first time that daytime napping was associated with increased CRP levels in a population-based middle- to older aged English cohort. The association between daytime napping and CRP levels was more pronounced among older people, and among those who spent extremes of time in bed at night. While the present study provides new insights into the biological effects of daytime napping, causality can not be established from an observational study. Daytime napping might be useful as an independent indicator of people who are at underlying health risk or

For peer review only - http://bmjopen.bmj.com/site/about/guidelines.xhtml

For peer review only

who suffer from disturbed sleep at night. Future physiological studies are needed to gain better understanding of the association.

Acknowledgement

We thank all study participants, general practitioners and the EPIC-Norfolk study team for their contribution.

Competing interests

None

Funding statement: The design and conduct of the EPIC-Norfolk study and collection and management of the data was supported by programme grants from the Medical Research Council UK (G9502233, G0300128) and Cancer Research UK (C865/A2883). YL is supported by the Cambridge Overseas Trust. FPC leads the Sleep Health & Society Programme at the University of Warwick supported, in part, by the University of Warwick RDF and IAS. It has received funding by the NHS National Workforce Projects and the Economic & Social Research Council (ES/K002910/1).

Authors' contributions

The work presented here was carried out in collaboration between all authors. YL and SA analyzed these data and wrote the manuscript with co-authors. YL, SA, NWJW, FPC, PGS, CB and KTK discussed the analysis, interpretation and presentation of these data. RL performed all data management and record linkage. RL and KTK are

For peer review only - http://bmjopen.bmj.com/site/about/guidelines.xhtml

For peer review only

on the management team of EPIC-Norfolk population study and contributed substantially to acquisition of data. KTK is a principal investigator in the EPIC-Norfolk study. All authors provided detailed comments on the draft, and revised the manuscript critically. All authors read and approved the final manuscript.

References

1 Milner C, Cote KA. Benefits of napping in healthy adults: impact of nap length, time of day, age, and experience with napping. *J Sleep Res* 2009;**18**:272–81.

2 Bursztyn M, Stessman J. The siesta and mortality: twelve years of prospective observations in 70-year-olds. *Sleep* 2005;**28**:345–7.

3 Jung K-I, Song C-H, Ancoli-Israel S, *et al.* Gender differences in nighttime sleep and daytime napping as predictors of mortality in older adults: the Rancho Bernardo study. *Sleep Med* 2013;**14**:12–9.

4 Tanabe N, Iso H, Seki N, *et al.* Daytime napping and mortality, with a special reference to cardiovascular disease: the JACC study. *Int J Epidemiol* 2010;**39**:233–43.

5 Xu Q, Song Y, Hollenbeck A, *et al.* Day napping and short night sleeping are associated with higher risk of diabetes in older adults. *Diabetes Care* 2010;**33**:78–83.

6 Leng Y, Wainwright NWJ, Cappuccio FP, *et al.* Daytime Napping and the Risk of All-Cause and Cause-Specific Mortality: A 13-Year Follow-up of a British Population. *Am J Epidemiol* 2014;**179**:1115–24.

7 Mullington JM, Simpson NS, Meier-Ewert HK, *et al.* Sleep loss and inflammation. *Best Pract Res Clin Endocrinol Metab* 2010;**24**:775–84.

8 Meier-Ewert HK, Ridker PM, Rifai N, *et al.* Effect of sleep loss on C-reactive protein, an inflammatory marker of cardiovascular risk. *J Am Coll Cardiol* 2004;**43**:678–83.

9 Jackowska M, Kumari M, Steptoe A. Sleep and biomarkers in the English Longitudinal Study of Ageing: associations with C-reactive protein, fibrinogen, dehydroepiandrosterone sulfate and hemoglobin. *Psychoneuroendocrinology* 2013;**38**:1484–93.

10 Patel SR, Zhu X, Storfer-Isser A, *et al.* Sleep duration and biomarkers of inflammation. *Sleep* 2009;**32**:200–4.

For peer review only - http://bmjopen.bmj.com/site/about/guidelines.xhtml

11 Dowd JB, Goldman N, Weinstein M. Sleep duration, sleep quality, and biomarkers of inflammation in a Taiwanese population. *Ann Epidemiol* 2011;**21**:799–806.

12 Grandner MA, Buxton OM, Jackson N, *et al.* Extreme sleep durations and increased C-reactive protein: effects of sex and ethnoracial group. *Sleep* 2013;**36**:769–779E.

13 Miller MA, Kandala N-B, Kivimaki M, *et al.* Gender differences in the cross-sectional relationships between sleep duration and markers of inflammation: Whitehall II study. *Sleep* 2009;**32**:857–64.

14 Taheri S, Austin D, Lin L, *et al.* Correlates of serum C-reactive protein (CRP)--no association with sleep duration or sleep disordered breathing. *Sleep* 2007;**30**:991–6.

15 Faraut B, Boudjeltia KZ, Dyzma M, *et al.* Benefits of napping and an extended duration of recovery sleep on alertness and immune cells after acute sleep restriction. *Brain Behav Immun* 2011;**25**:16–24.

16 Dijk DJ, Duffy JF, Czeisler CA. Contribution of circadian physiology and sleep homeostasis to age-related changes in human sleep. *Chronobiol Int* 2000;**17**:285–311.

17 Foley D, Vitiello M, Bliwise D, *et al.* Frequent napping is associated with excessive daytime sleepiness, depression, pain, and nocturia in older adults. *Am J Geriatr Psychiatry* 2007;**15**:344–50.

18 Ohayon MM, Carskadon M a, Guilleminault C, *et al.* Meta-analysis of quantitative sleep parameters from childhood to old age in healthy individuals: developing normative sleep values across the human lifespan. *Sleep* 2004;**27**:1255–73.

19 Leng Y, Wainwright NWJ, Cappuccio FP, *et al.* Self-reported sleep patterns in a British population cohort. *Sleep Med* 2014;**15**:295–302.

20 Suarez EC. Self-reported symptoms of sleep disturbance and inflammation, coagulation, insulin resistance and psychosocial distress: evidence for gender disparity. *Brain Behav Immun* 2008;**22**:960–8.

21 Day N, Oakes SA, Luben R, *et al.* EPIC-Norfolk: study design and characteristics of the cohort. *Br J Cancer* 1999;**80**:95–103.

22 Ahmadi-Abhari S, Luben RN, Wareham NJ, *et al.* Distribution and determinants of C-reactive protein in the older adult population: European Prospective Investigation into Cancer-Norfolk study. *Eur J Clin Invest* 2013;**43**:899–911.

23 Wareham NJ, Jakes RW, Rennie KL, *et al.* Validity and repeatability of a simple index derived from the short physical activity questionnaire used in the

For peer review only

For peer review only - http://bmjopen.bmj.com/site/about/guidelines.xhtml

European Prospective Investigation into Cancer and Nutrition (EPIC) study. *Public Health Nutr* 2003;**6**:407–13.

24 Heslop P, Smith GD, Metcalfe C, *et al.* Sleep duration and mortality: The effect of short or long sleep duration on cardiovascular and all-cause mortality in working men and women. *Sleep Med* 2002;**3**:305–14.

25 Hublin C, Partinen M, Koskenvuo M, *et al.* Sleep and mortality: a population-based 22-year follow-up study. *Sleep* 2007;**30**:1245–53.

26 Bursztyn M. The siesta in the elderly: risk factor for mortality? *Arch Intern Med* 1999;**159**:1582–6.

27 Cohen-Mansfield J, Perach R. Sleep duration, nap habits, and mortality in older persons. *Sleep* 2012;**35**:1003–9.

28 Ruggiero JS, Redeker NS. Effects of Napping on Sleepiness and Sleep-Related Performance Deficits in Night-Shift Workers: A Systematic Review. *Biol Res Nurs* 2014;**16**:134–42.

29 Tassi P, Muzet A. Sleep inertia. *Sleep Med Rev* 2000;**4**:341–53.

30 Lavie P. Self-reported sleep duration--what does it mean? *J Sleep Res* 2009;**18**:385–6.

31 Wium-Andersen MK, Ørsted DD, Nielsen SF, *et al.* Elevated C-reactive protein levels, psychological distress, and depression in 73, 131 individuals. *JAMA Psychiatry* 2013;**70**:176–84.

32 Allin KH, Bojesen SE, Nordestgaard BG. Baseline C-reactive protein is associated with incident cancer and survival in patients with cancer. *J Clin Oncol* 2009;**27**:2217–24.

33 Kaptoge S, Di Angelantonio E, Lowe G, *et al.* C-reactive protein concentration and risk of coronary heart disease, stroke, and mortality: an individual participant meta-analysis. *Lancet* 2010;**375**:132–40.

34 Meier-Ewert HK, Ridker PM, Rifai N, *et al.* Absence of diurnal variation of C-reactive protein concentrations in healthy human subjects. *Clin Chem* 2001;**47**:426–30.

35 Campos H, Siles X. Siesta and the risk of coronary heart disease: results from a population-based, case-control study in Costa Rica. *Int J Epidemiol* 2000;**29**:429–37.

36 Lam K-BH, Jiang CQ, Thomas GN, *et al.* Napping is associated with increased risk of type 2 diabetes: the Guangzhou Biobank Cohort Study. *Sleep* 2010;**33**:402–7.

For peer review only - http://bmjopen.bmj.com/site/about/guidelines.xhtml

37 Naska A, Oikonomou E, Trichopoulou A, *et al.* Siesta in healthy adults and coronary mortality in the general population. *Arch Intern Med* 2007;**167**:296–301.

38 Gao J, Huang X, Park Y, *et al.* Daytime napping, nighttime sleeping, and Parkinson disease. *Am J Epidemiol* 2011;**173**:1032–8.

39 Foley D, Monjan A, Masaki K, *et al.* Daytime sleepiness is associated with 3-year incident dementia and cognitive decline in older Japanese-American men. *J Am Geriatr Soc* 2001;**49**:1628–32.

40 Chen H, O'Reilly EJ, Schwarzschild M a, *et al.* Peripheral inflammatory biomarkers and risk of Parkinson's disease. *Am J Epidemiol* 2008;**167**:90–5.

41 Ferrie JE, Kivimäki M, Akbaraly TN, *et al.* Associations Between Change in Sleep Duration and Inflammation: Findings on C-reactive Protein and Interleukin 6 in the Whitehall II Study. *Am J Epidemiol* 2013;**178**:956–61.

42 Mullington JM, Haack M, Toth M, *et al.* Cardiovascular, inflammatory, and metabolic consequences of sleep deprivation. *Prog Cardiovasc Dis* 2009;**51**:294–302.

43 Chiu J-J, Usami S, Chien S. Vascular endothelial responses to altered shear stress: pathologic implications for atherosclerosis. *Ann Med* 2009;**41**:19–28.

44 Liukkonen T, Räsänen P, Ruokonen A, *et al.* C-reactive protein levels and sleep disturbances: observations based on the Northern Finland 1966 Birth Cohort study. *Psychosom Med* 2007;**69**:756–61.

45 Svensson M, Venge P, Janson C, *et al.* Relationship between sleep-disordered breathing and markers of systemic inflammation in women from the general population. *J Sleep Res* 2012;**21**:147–54.

For peer review only

For peer review only - http://bmjopen.bmj.com/site/about/guidelines.xhtml

Figure 1: Serum levels of C-reactive protein by napping and time in bed *

* Values are geometric mean CRP (mg/l) across categories adjusted for age, body mass index, physical activity, smoking, alcohol intake, social class, education, marital status, major depressive disorder, self-reported health, pre-existing diseases, systolic blood pressure, hypnotic drug use, and in women only, postmenopausal hormone replacement therapy. The sleep measures were mutually adjusted. Vertical bars represent 95% confidence intervals.

For peer review only

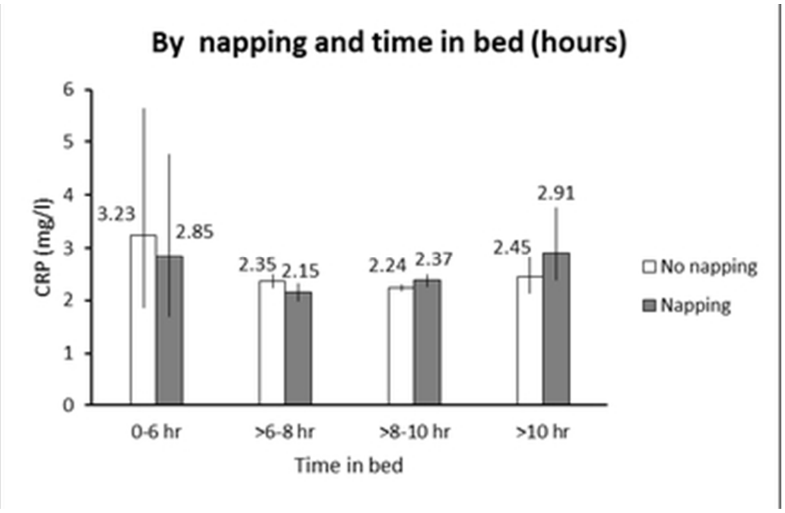

33x21mm (300 x 300 DPI)

STROBE 2007 (v4) checklist of items to be included in reports of observational studies in epidemiology*
Checklist for cohort, case-control, and cross-sectional studies (combined)

Section/Topic	Item #	Recommendation	Reported on page #
**Title and abstract**	1	(*a*) Indicate the study's design with a commonly used term in the title or the abstract	1
		(*b*) Provide in the abstract an informative and balanced summary of what was done and what was found	2
**Introduction**			
Background/rationale	2	Explain the scientific background and rationale for the investigation being reported	5
Objectives	3	State specific objectives, including any pre-specified hypotheses	6
**Methods**			
Study design	4	Present key elements of study design early in the paper	6-7
Setting	5	Describe the setting, locations, and relevant dates, including periods of recruitment, exposure, follow-up, and data collection	6-7
Participants	6	(*a*) *Cohort study*—Give the eligibility criteria, and the sources and methods of selection of participants. Describe methods of follow-up<br>*Case-control study*—Give the eligibility criteria, and the sources and methods of case ascertainment and control selection. Give the rationale for the choice of cases and controls<br>*Cross-sectional study*—Give the eligibility criteria, and the sources and methods of selection of participants	6-7
		(*b*) *Cohort study*—For matched studies, give matching criteria and number of exposed and unexposed<br>*Case-control study*—For matched studies, give matching criteria and the number of controls per case	
Variables	7	Clearly define all outcomes, exposures, predictors, potential confounders, and effect modifiers. Give diagnostic criteria, if applicable	7-8
Data sources/ measurement	8*	For each variable of interest, give sources of data and details of methods of assessment (measurement). Describe comparability of assessment methods if there is more than one group	7
Bias	9	Describe any efforts to address potential sources of bias	8-9
Study size	10	Explain how the study size was arrived at	8
Quantitative variables	11	Explain how quantitative variables were handled in the analyses. If applicable, describe which groupings were chosen and why	8-9
Statistical methods	12	(*a*) Describe all statistical methods, including those used to control for confounding	9
		(*b*) Describe any methods used to examine subgroups and interactions	9
		(*c*) Explain how missing data were addressed	8
		(*d*) *Cohort study*—If applicable, explain how loss to follow-up was addressed<br>*Case-control study*—If applicable, explain how matching of cases and controls was addressed	8

For peer review only

		Cross-sectional study—If applicable, describe analytical methods taking account of sampling strategy	
		(*e*) Describe any sensitivity analyses	9
**Results**			
Participants	13*	(a) Report numbers of individuals at each stage of study—eg numbers potentially eligible, examined for eligibility, confirmed eligible, included in the study, completing follow-up, and analysed	10
		(b) Give reasons for non-participation at each stage	
		(c) Consider use of a flow diagram	
Descriptive data	14*	(a) Give characteristics of study participants (eg demographic, clinical, social) and information on exposures and potential confounders	10
		(b) Indicate number of participants with missing data for each variable of interest	13
		(c) *Cohort study*—Summarise follow-up time (eg, average and total amount)	
Outcome data	15*	*Cohort study*—Report numbers of outcome events or summary measures over time	
		Case-control study—Report numbers in each exposure category, or summary measures of exposure	
		Cross-sectional study—Report numbers of outcome events or summary measures	8
Main results	16	(*a*) Give unadjusted estimates and, if applicable, confounder-adjusted estimates and their precision (eg, 95% confidence interval). Make clear which confounders were adjusted for and why they were included	12
		(*b*) Report category boundaries when continuous variables were categorized	
		(*c*) If relevant, consider translating estimates of relative risk into absolute risk for a meaningful time period	
Other analyses	17	Report other analyses done—eg analyses of subgroups and interactions, and sensitivity analyses	15
**Discussion**			
Key results	18	Summarise key results with reference to study objectives	16
Limitations	19	Discuss limitations of the study, taking into account sources of potential bias or imprecision. Discuss both direction and magnitude of any potential bias	16-17
Interpretation	20	Give a cautious overall interpretation of results considering objectives, limitations, multiplicity of analyses, results from similar studies, and other relevant evidence	17-19
Generalisability	21	Discuss the generalisability (external validity) of the study results	17
**Other information**			
Funding	22	Give the source of funding and the role of the funders for the present study and, if applicable, for the original study on which the present article is based	21

*Give information separately for cases and controls in case-control studies and, if applicable, for exposed and unexposed groups in cohort and cross-sectional studies.
Note: An Explanation and Elaboration article discusses each checklist item and gives methodological background and published examples of transparent reporting. The STROBE checklist is best used in conjunction with this article (freely available on the Web sites of PLoS Medicine at http://www.plosmedicine.org/, Annals of Internal Medicine at http://www.annals.org/, and Epidemiology at http://www.epidem.com/). Information on the STROBE Initiative is available at www.strobe-statement.org.

For peer review only
